# How Force Might Activate Talin's Vinculin Binding Sites: SMD Reveals a Structural Mechanism

**DOI:** 10.1371/journal.pcbi.0040024

**Published:** 2008-02-15

**Authors:** Vesa P Hytönen, Viola Vogel

**Affiliations:** Laboratory of Biologically Oriented Materials, Department of Materials, Swiss Federal Institute of Technology Zurich (ETH Zurich), Zürich, Switzerland; Adolf-Butenandt-Institut, Germany

## Abstract

Upon cell adhesion, talin physically couples the cytoskeleton via integrins to the extracellular matrix, and subsequent vinculin recruitment is enhanced by locally applied tensile force. Since the vinculin binding (VB) sites are buried in the talin rod under equilibrium conditions, the structural mechanism of how vinculin binding to talin is force-activated remains unknown. Taken together with experimental data, a biphasic vinculin binding model, as derived from steered molecular dynamics, provides high resolution structural insights how tensile mechanical force applied to the talin rod fragment (residues 486–889 constituting helices H1–H12) might activate the VB sites. Fragmentation of the rod into three helix subbundles is prerequisite to the sequential exposure of VB helices to water. Finally, unfolding of a VB helix into a completely stretched polypeptide might inhibit further binding of vinculin. The first events in fracturing the H1–H12 rods of talin1 and talin2 in subbundles are similar. The proposed force-activated α-helix swapping mechanism by which vinculin binding sites in talin rods are exposed works distinctly different from that of other force-activated bonds, including catch bonds.

## Author Summary

For cell survival, most eukaryotic cells need to be mechanically anchored to their environment. This is done by transmembrane proteins, including integrins, which externally bind to the extracellular matrix and on the cell interior to the contractile cytoskeleton via scaffolding proteins. One essential scaffolding protein is talin, which binds to integrins via its head and to the cytoskeletal filament f-actin via its rodlike tail. As cells apply tensile forces to newly formed adhesion sites, proteins that are part of such force-bearing networks get stretched and might change their structure and thus function. One of many proteins that are recruited to newly formed adhesions is vinculin, and vinculin recruitment is upregulated by tensile mechanical force—but how? Since talin's vinculin binding sites are buried in its native structure, we used steered molecular dynamics here to derive a high resolution structural model of how tensile mechanical forces might activate talin's vinculin binding sites. Once tensile forces break up the talin rod into helix subbundles, an event that we find here to constitute the main energy barrier, we propose how the strain-induced gradual exposure of the vinculin-binding helices finally allows for their activation and enables helix swapping with the vinculin head.

## Introduction

**Figure 1:**
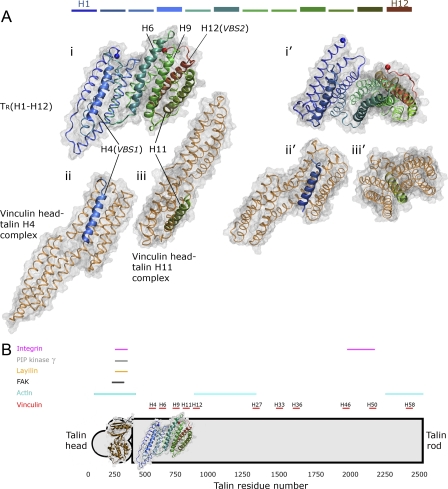
Structures of the α-Helix Bundle of Talin and of Its Vinculin Binding (VB) Helices in Complex with the Vinculin Head (A) The talin rod fragment H1–H12, which contains five VB helices. The coloring is according to residue numbers (blue, N-terminus; red, C-terminus; colors of helices are shown at the top of the figure). The VB helices H4, H6, H9, H11, and H12 are shown in bold cartoon models, and the rest of the structure is presented by narrow ribbons. The molecular surface is presented in gray (1.4 Å scanning probe used). Terminal Cα-atoms are shown as spheres. (i) Talin fragment H1–H12 (PDB 1XWX). (ii) Vinculin head domain (V_H_) bound to talin helix H4 (VBS1) (PDB 1SYQ). V_H_ (brown ribbon model) is aligned onto the talin rod fragment according to talin H4 (blue cartoon model). (iii) Vinculin head domain bound to talin H11 (VBS4; PDB 1ZVZ) aligned onto the talin rod according to H11. (i'–iii') are rotated views of (i–iii). (B) Overall schematic structure of talin indicating the location of the VB helices and other protein binding sites (integrin [3,12], layilin [5], PIP kinase γ [4], focal adhesion kinase [FAK] [5], actin [11], and vinculin [10]). The structurally unresolved talin head and rod domains are shown in gray overlaid with the known structures. Few trypsin-sensitive sites in the chicken T_R_ have been determined, maybe indicating either some local looseness of the packing in the distant C-terminal rod region even under equilibrium conditions, or high flexibility in particular loops connecting helices (known cleaving sites in T_R_: residue 1653, between H36 and H37; 1804, in H41) [57].

Talin physically links integrins to the contractile cytoskeleton [1,2]. The talin head (T_H_), residues 1–432, has binding sites for integrin β-tails [3], PIP kinase γ [4], focal adhesion kinase (FAK) [5], layilin [5] and actin [6] (Figure 1B). Binding of the talin head to the cytoplasmic tail of β-integrins can cause integrin activation [7]. The 60 nm long talin rod (T_R_), residues 433-2541, is composed of bundles of amphiphatic α-helices [8,9] (Figure 1). The talin rod contains up to eleven vinculin binding sites [10] (Figures 1B and S1), including five located within fragment H1–H12, residues 486–889, which is studied here (Figure 1). All of these five binding sites are buried inside helix bundles (native talin shows considerably lower affinity to vinculin compared to peptide fragments isolated from talin). In addition to the VBSs, the talin rod has binding sites for actin [11] and for integrins [12].

Upon cell adhesion, talin rapidly accumulates in focal contacts prior to vinculin recruitment [13]. Only talin, but not vinculin or paxillin, is recruited to the clustered integrins, if β3 integrins are activated not by ligand binding but by manganese (Mn^2+^), i.e., in cases where integrin activation occurs without the application of force and integrin is thus not part of a force-bearing protein network [14]. Indeed, the recruitment of vinculin to cell adhesion sites has been shown to be force-dependent [15–19]. Vinculin recruitment strengthens cell adhesions [20], and reduces focal adhesion turnover [21]. In focal adhesions, vinculin is in an activated state while it is autoinhibited in the surrounding cytoplasm [22]. Important to the model proposed here is that vinculin activation can also be induced in solution by isolated talin peptides containing VBSs [23].

As talin, vinculin is an alpha-helical protein sharing considerable structural similarity. The x-ray structure of full-length vinculin [PDB: 1TR2] reveals three head domains, V_H_, V_H_2, and V_H_3, that are connected to the tail domains, V_T_2 and V_T_, via a flexible, proline-rich linker. Vinculin's talin binding site is located in the V_H_ head domain, which is composed of two four-helix bundles sharing one helix [24] (Figure 1A). Since V_H_ can bind to V_T_, thereby confining the molecule to an autoinhibitory closed conformation, vinculin activation is needed to increase its affinity to talin and actin [25,26].

While it was suggested that vinculin recruitment to focal adhesions is force-regulated [15–19], no high resolution structural mechanism has been derived experimentally by how force can activate talin's buried vinculin binding sites. What is known, however, is that fragmentation of the talin rod into smaller isolated helix bundles can activate talin's VBS [27], and that point mutations that destabilize helix-helix interactions can have a similar activating effect [27]. Finally, vinculin binding to talin's VBS can cause significant loss of secondary structure of the adjacent non-VB-helices [28,29]. Alternatively, it has been hypothesized that binding of PIP2 lipids to the talin rod may also lead to the activation of VB sites as the presence of PIP2 slightly increased the binding of vinculin to talin [30]. No mechanism has been suggested though how activation by PIP2 could directly explain force-upregulated vinculin recruitment to newly formed cell adhesion sites.

To establish a potential mechanism of force-regulated activation of talin's VBSs, we used steered molecular dynamics (SMD) to study the force-induced conformational changes in the N-terminal talin rod fragment H1–H12 (a model created by merging two experimentally determined overlapping structures covering residues 486–889 [28]). This computational method is valuable since no experimental techniques are available to obtain high resolution information how the structure of proteins is changed when they are mechanically stretched. Access to such information is essential to learn how force might activate proteins by switching their structure-function relation (as reviewed in [31,32]). Therefore, known equilibrium structures of the talin rod were solvated here computationally in a box filled with explicit water molecules. Conducting the simulations in the presence of explicit water rather than in an unstructured dielectric medium is important, since the access of free water molecules to often buried force-bearing hydrogen bonds or salt bridges can regulate mechanical stability [31–34]. After equilibration for 1 ns, constant force was applied to either the termini of the rod fragment, or alternatively to putative force-bearing interfaces which might stabilize this rod fragment in the intact talin molecule. We then characterized the structural changes that lead to the sequential exposure of the vinculin binding sites. A mechanism of how stretching of the talin rod might activate vinculin binding is proposed.

## Results

**Figure 2:**
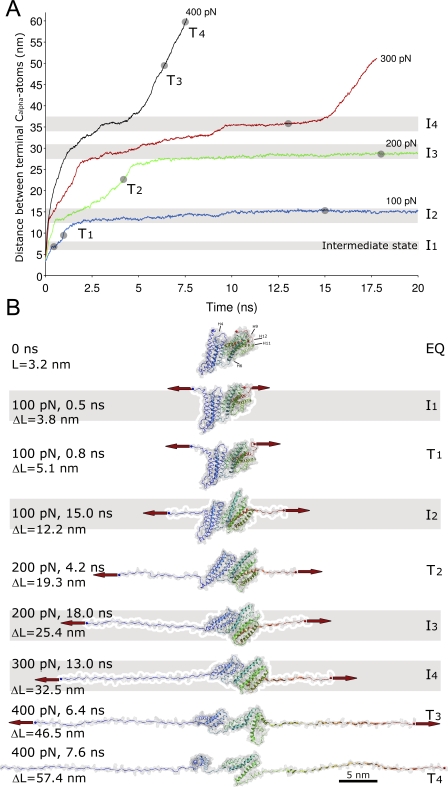
Sequential Unfolding Trajectories and Associated Structures of the Talin Rod H1–H12 Extended under Constant Force Applied to Terminal Cα-atoms (A) Extension-time plots for different constant force pulls, from 100–400 pN. Plateaus in the extension-time curves indicate the existence of multiple intermediate states (I_1_–I_4_). Simulations are carried out using the program NAMD. The SMD simulation was started after 1 ns of equilibration at a temperature of 310 K and 1 atm pressure. The explicit water model TIP3 was used. (B) Intermediate states (I_1–4_, gray bars) and transitional snapshots (T_1–4_) seen at indicated time points in the unfolding trajectories in (A).

**Figure 3:**
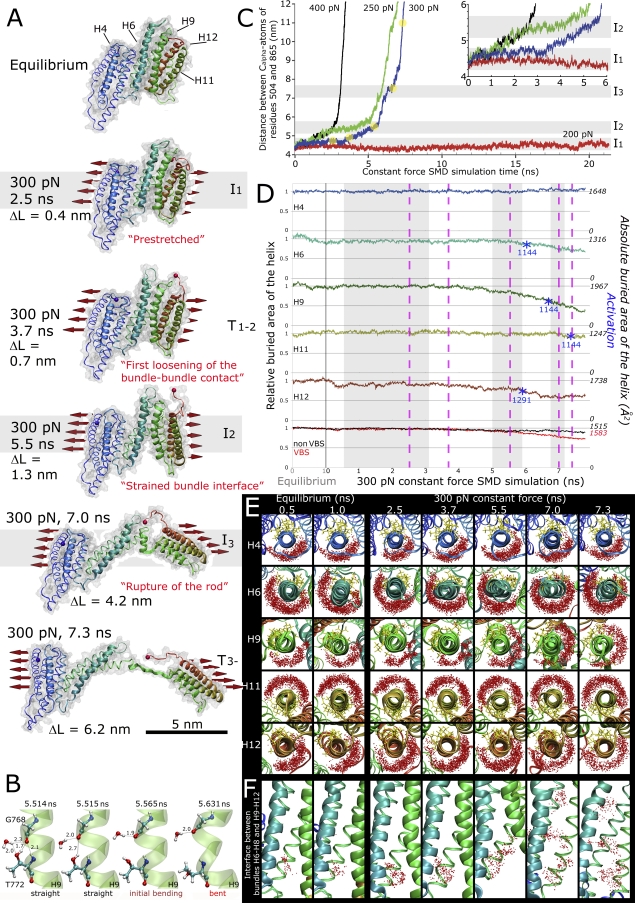
Fragmentation of the Talin Rod into α-Helix Subbundles Leads to the Sequential Exposure of the Vinculin Binding Helices (VB Helices) (A) Sequential structural snapshots of the mechanically strained talin H1–H12 rod. Three intermediate states are observed (I_1_, I_2_, I_3_). The pulling force of 300 pN was used in the depicted SMD simulation. The extension, i.e., the increase in the length of the H1–H12 as compared to the equilibrium state which measured 3.2 nm, is shown as (ΔL). Key transitional unfolding events detected in this simulation as H9–H12 separates from rest of the protein are shown in T_1_ and T_2_. (B) SMD simulations showed a transient bending of helix H9 at the onset of breaking the H1–H12 bundle into two pieces. The bending occurred at residue Thr772, which initially formed intrahelix bonds with Gly768:O with both the backbone nitrogen and the side chain oxygen. The hydrophilic side chain in Thr772 attracts water molecules that enter the helix bundle cleft when the talin rod is strained. The waters soon reach the helix backbone (5.514 ns), and then compete and attack the backbone hydrogen bond initially formed between Thr772 and Gly768, finally leading to the bending of the helix (5.631 ns). (C) Constant force extension-time plots when force is distributed over the length of the terminal helices H1 and H12 (200, 250, 300, or 400 pN). The distance between Cα-atoms of residues 504 and 865 is given. The extensions of the structural snapshots shown in (B) are indicated in the extension time-plots in (C). (D) Change in the buried surface area of the VB helices during equilibration and when extended under 300 pN force calculated for the blue 300 pN trace shown in (C). The buried areas are shown normalized to the average buried area obtained during equilibration. The lowest graph shows the average buried area of the VB helices (red) and of other helices (nonVB, black). The dotted pink lines give the times at which the non-equilibrium structural snapshots in (B) were taken. The respective points of “activation”, i.e., when the buried areas of helices H6, H9, H11, and H12 in talin equal the experimentally found buried areas of isolated talin helices in complex with the vinculin head, are given as blue asterisks. For H6 and H9, the buried area determined for the H11-vinculin complex is used as a reference because there is no available structure of those helices in complex with vinculin. The buried area of H4 was higher than the buried area of the V_H_-H4 complex for the whole simulation period. The buried area of a helix was calculated by measuring the solvent accessible surface area (SASA) of the helix alone and subtracting the SASA of the helix embedded in the protein by using the program VMD. The scanning probe used was 1.4 Å. (E) Top views of the VB helices along the helix axes. Water molecules (red dots) located within 5 Å of the VB helix core (10–13 residues in the middle of the helix) from 20 frames over 100 ps time window are plotted together with the protein structure at different time points. The side chains of 10 most conserved residues in VBS helices [10], according to the consensus sequence LxxAAxxVAxALxxLLxxA are shown in a yellow stick representation. (F) Side views showing how water penetrates into the interface between the subbundles H1–H8 and H9–H12. The water molecules located in the vicinity of residues Leu716, Leu736, and Gly740 are plotted over a 100 ps time window (20 frames). The frames in the trajectory are aligned according to H8.

Since the talin is anchored to integrins via the head domain, while actin binding sites exist downstream at the C-terminus of the H1–H12 bundle (see Figure 1B), the force vector acting between the membrane-bound integrins and the cytoskeleton are likely transmitted through talin along the long axis of the H1–H12 rod. Unclear is, however, whether the force in full length talin acts directly on the two terminal atoms of the H1–H12 bundle, or given the tilt of the helices with respect to the long rod axis of talin [27–29], whether it is distributed over force-bearing interfaces formed with the adjacent domains. The H1 helix might form a force-bearing interface with the talin head (the structure of the linker region connecting T_H_ and T_R_ is not known), while the H12 helix might be tightly packed against the rest of the structurally unresolved C-terminal talin rod (residues 890-2541). We thus conducted two sets of constant force simulations where the force is either applied locally to the two terminal Cα-atoms of the H1–H12 bundle, or is distributed along the length of the terminal helices, H1 and H12, to mimic the existence of such putative force-bearing interfaces. For both cases, we analyzed how stretching the talin rod with constant force would alter its structure. The little resistance to strainnig the bundle is due to the unfolding of secondary structure and leads to a rapid molecular extension with time. If a major energy barrier has to be overcome to allow the further unfolding of the rod, the protein domain will pause to extend as indicated by a plateau region in the extension-time plots (Figures 2 and 3). We thus asked how the structure of the H1–H12 bundle is changed with time as we pull on the rod with constant force, and whether one major event leads to the complete disintegration of the rod or whether *intermediate states (I)* exist separated by energy barriers. Please note that the lifetimes of intermediate states increase as the forces are lowered to physiologically relevant forces (for further discussion see [31,32,35,36]). Due to limited computational resources, however, the simulations shown here can only be run for several nanoseconds and higher forces are required to trigger the events described. For other molecular systems, however, it has been shown that these constant force approaches can correctly predict the positions of major energy barriers along physiologically significant unfolding pathways [31,32,34,37,38].

### The Terminal Helices Tend To Unfold Easily if the Force Is Applied Directly to the Terminal Atoms of the H1–H12 Bundle

When applying various constant forces *locally* to the terminal atoms of the H1–H12 bundle (100 pN, 200 pN, 300 pN, and 400 pN), the intial end-to-end distance of the talin rod H1–H12 of 3.2 nm increases rapidly with time due to progressive unfolding (loss of secondary structure) of the terminal helices (Figure 2B). The ease by which they unravel even at low forces indicates that relatively little energy is required to sequentially break the backbone hydrogen bonds, turn-by-turn, that stabilize their helical secondary structure [39,40]. As shown in Figure 2A, a first short-lived plateau can be seen that has a lifetime of less than 1 ns if we pull at 100 pN (*Intermediate State I_1_*). Just little activation is then needed to begin the unfolding of both of the terminal helices, H1 and H12, which leads to an extension with respect to the resting length of altogether 15 nm, until a second plateau is reached (*Intermediate State I_2_*)*.* Notice though that both helices do not initially unfold completely. Only the N-terminal half of H1 (residues 499–503) is unraveled in state *I_2,_* due to a bent in H1 at Ser502. The bending site seems to be defined by the side chain of residue Ser502, which competes for the backbone hydrogen bond between the Ile499 and Met503 (see Figure 3B for presentation of such an event). Similarly for the H12, which is a vinculin binding helix, its C-terminal residues 874–879 are unraveled while its N-terminal part of the helix remains buried in the helix bundle.

Further extension results from the sequential turn-by-turn unraveling of the N-terminal end of H1 (D_2_; in all of 8 local force simulations; Figure 2), and the H12 by ∼3 additional turns (*Intermediate State I_3_*) which leads to a total extension of 28 nm while pausing in the third plateau in Figure 2A. This intermediate *I_3_*, which is seen to last for ∼15 ns at 200 pN, is characterized by a completely unraveled H1, whereas residues 868–879 of H12 are unraveled while residues 849–867 of H12 are still in a helical conformation and in tight contact with the rest of the H9–H12 bundle. At higher forces, we see a rapid transitioning into *I_4_ (multiple unraveled helices)* where both H2 (∼8 turns unraveled) and H12 (∼6 turns unraveled) are mostly unraveled. *I_4_* is characterized by an extension of 35–36 nm. Once the major energy barrier to rupture *I_4_* is passed (as described below), the remaining bundle H2–H11 rapidly breaks down into the bundles H2–H5, H6–H8 and H9–H12 (T_4_ in Figure 2). Each of these three helix bundles unfolds independently from each other at later times as discussed below. Note that the two vinculin binding helices, H11 and H12, are already completely unraveled when T_4_ is reached, i.e., they convert early on into an extended polypeptide chain held under tension.

### If the Tensile Force Is Distributed along the Length of the Terminal Helices (Mimicking Force-Bearing Interfaces), the Talin Rod Is Mechanically More Stable

To assess whether the just described intermediates are characteristic only for an isolated talin fragment where force is applied to the terminal atoms, or also for the case where force is distributed over the cross-section of the talin rod, we ran constant force simulations in which we distributed the force (200 pN, 250 pN, 300 pN, 400 pN) along the C_α_-atoms of the terminal helices H1 and H12 as shown in Figure 3. Additional constant velocity simulations were carried out under otherwise similar conditions, but by applying force distributed along the C-terminal H12 (see Methods) and harmonically constraining H1 (Figure S2, only stimulation with harmonical constrained helix).

**Figure 4:**
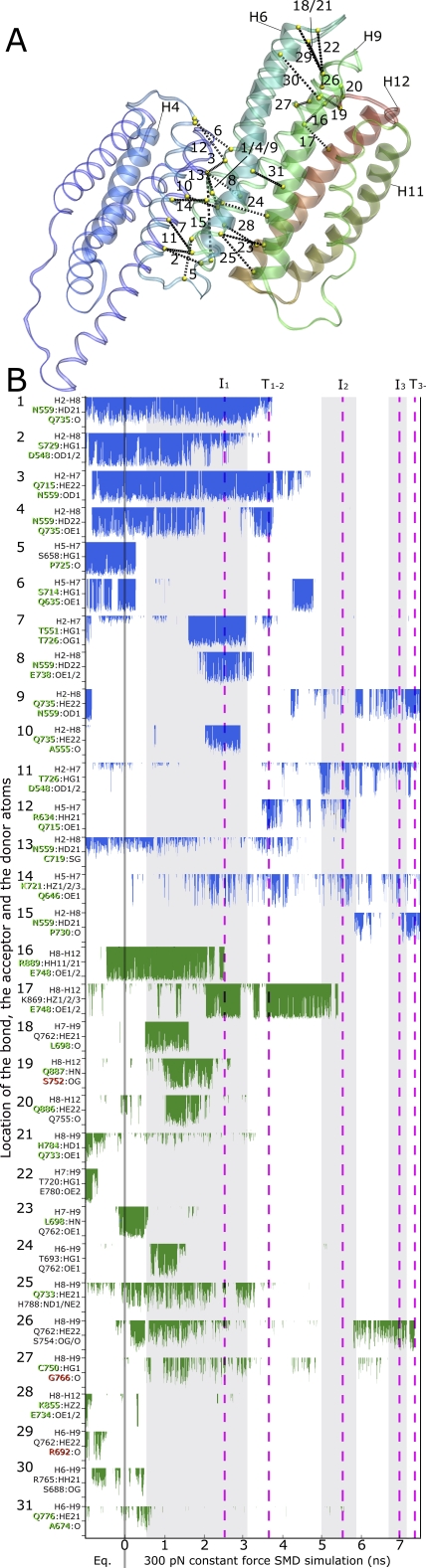
Side Chain Hydrogen Bonds Formed between the α-Helix Subbundles, H1–H5, H6–H8, H9–H12 in Talin1 as Identified in the Simulations (A) Location of interbundle side chain hydrogen bonds is shown schematically in the equilibrated structure (Connection [black] of the Cα-atoms of the interacting residues [shown in yellow]). (B) Stability of interbundle hydrogen bonds: the bond length fluctuations are given at a scale of 2-4 Å, beyond which the bond is considered broken. In all the cases where amino acid side chains provide multiple donors or acceptors, the shortest of the bonds formed is shown. Multiple bonding partners are listed. The fluctuations of bonds connecting the helix bundles of H1–H5 to H6–H8 are shown in blue, while the ones connecting the H6–H8 to H9–H12 bundles are shown in green. The fully conserved residues among the talin1 and talin2 from human, mouse, and chicken (Figure S1) are written in green, and non-conserved residues in red.

Distributing the force over a putative interface had a major stabilizing impact on the resulting unfolding trajectories and the forces needed to activate the VBSs (Figures 2 and 3): The most notable difference is that the talin rod H1–H12 could withstand considerable higher forces before breaking apart (Figure 3C), stably exceeding our simulation window of 20 ns in a prestretched intermediate state *I_1_* (Figure 3) if pulled with a constant force (200 pN). Earlier, this force had resulted in the unraveling of the terminal helices within the first 5 ns (Figure 2A) if force was only applied to the terminal atoms. Distributing the force over the terminal helices thus stabilized the entire H1–H12 bundle against force-induced breakage and terminal helix unraveling: unfolding of H1 was not detected as a first major unfolding event even at higher forces (7 simulations, 250–400 pN). Plotting the sequence by which hydrogen bonds between the helix bundles broke (Figure 4) reveals that straining the talin rod leads to the appearance of some new hydrogen bonds (for example bond 8 between residues Asn559 and Glu738) while others break (for example bond 5 between residues Ser658 and Pro725).

When H1–H12 is stretched with a constant force of 250–400 pN distributed over the terminal helices, interhelical bundle contacts start to loosen up after a few ns leading to a slight opening of the interface between the bundles H9–H12 and H1–H8 (*I_2_*). This passage leads to the breakdown of some hydrogen bonds in the interface between H1–H8 and H9–H12, namely between residues Gln887-Ser752, Gln886-Gln755, His784-Glu733, His788-Gln733 (referred to as bonds 19, 20, 21, 25 in Figure 4). Also the bonding between bundles H1–H5 and H6–H8 is weakened significantly by breakdown of hydrogen bonds between residues Asn559-Gln735, Ser729-Asp548, Gln715-Asn559 (referred to as bonds 1, 2, 3 in Figure 4). Further pulling results in a gradual opening of the interface between the bundles and water starts to penetrate between the helices (Figure 3E). The stress applied leads finally to a bending of H9 around residue 770 due to breakage of the backbone hydrogen bond formed between residues Gly768 and Thr772 (Figure 3B). After this, H9 stays attached to H1–H8 on the N-terminal end during the short-lived intermediate state *I_3_* seen in many but not in all the simulations (5 of 7 simulations 250–400 pN). After passing the *I_3_* intermediate, a rapid disintegration of the talin rod is seen (T_3−_) which then allows for the sequential unfolding of the now separated bundles.

### Talin Rod Fragmentation Eliminates Force-Bearing Interfaces Which Then Weakens the Mechanical Stabilities of Its Fragments

**Figure 5:**
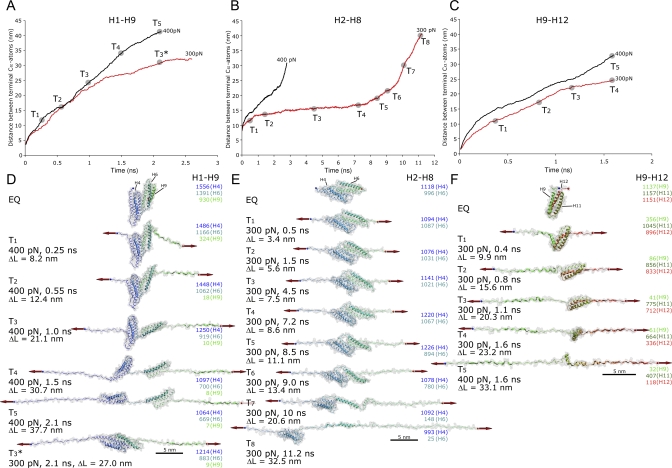
Unfolding Trajectories of the Individual Talin Rod Subbundles Once the talin rod is fragmented, the force will be transmitted via the terminal ends of the helices. Constant forces of 300 and 400 pN were thus applied to the termini of the fragments H1–H9 (A) and (D); H2–H8 (B) and (E); and H9–H12 (C) and (F) after 1 ns of equilibration. The distance between the terminal Cα-atoms of the fragments are plotted over time. The starting end-to-end distances of the terminal atoms of these helix bundles prior to stretching them were 3.6 nm (H1–H9), 7.9 nm (H2–H8), and 1.5 nm (H9–H12). (A,D) Force applied to the H1–H9 fragment first leads to the unfolding of H9 (T_1_) and is then followed by the unfolding of H1 (T_2_). Further pulling with 400 pN results in the separation of bundles H2–H5 and H6–H8 from each other (T_2_–T_3_). Then, the C-terminal bundle H6–H8 is the first to be unraveled (T_5_). The 300 pN simulation does not lead to a separation of bundles within 2.7 ns (T_3_*) and shows similarity with T_3_ of the H2–H8 fragment (B) and (E). (B,E) The 300 pN unfolding trajectory of the H2–H8 bundle not truncated along the “natural” interfaces shows sequential unfolding at the ends of the molecule (T_1_–T_3_). 400 pN force applied results in faster unfolding. The C-terminal part unfolds more easily indicating the lower stability of H6–H8 compared to H2–H5 (T_7_, T_8_). (C,F) The H9–H12 fragment shows only negligible resistance against applied force even if pulled at 300 pN force.

Common to all simulations is the break-up of the talin rod, H1–H12, into smaller helix bundles that have far smaller mechanical stabilities. We observed that the first split occurred between H1–H8 and H9–H12 (6 times in 7 simulations where constant force is applied over terminal helices), after which H1–H8 splits into H1–H5 and H6–H8 (Figures 2, 3, and 5). In the other case observed only once in seven simulations, H1–H5 separated first followed by the H9–H12 separation. The break-up into these well-defined bundles as a major event is most clearly seen if the force is distributed over the terminal helices (Figure 3). Once the force-bearing interfaces are broken apart, the bundles have no stabilizing effects upon each other any longer. Consequently, the force is thus transmitted at these later times through the N- and C-terminal atoms of the resulting helix bundles. We thus simulated separately the unfolding of the helix bundles H1–H9 (crystallographically determined structure of talin rod fragment, PDB 1SJ8), H2–H8 (intermediate found in terminal atom pulls), and H9–H12 (intermediate seen only in force-bearing interface simulations). The starting end-to-end distances of the terminal atoms of these helix bundles prior to stretching were 3.6 nm (H1–H9), 7.9 nm (H2–H8) and 1.5 nm (H9–H12). Most noticeable when looking at all their unfolding pathways (Figure 5) is the lack of well pronounced plateaus and thus clearly distinguishable intermediate states, especially in the case of H9–H12. If pulled apart at 300 pN, the H1–H12 bundle breaks into bundles H1–H8 and H9–H12 after 7 ns (Figure 3). This indicates that different structural changes are happening in parallel at different positions.

Furthermore, when simulating the mechanical stabilities of alternate talin rod fragments that were not truncated along a ‘natural' bundle-bundle interface, for example simulating H1–H9 instead of H2–H8, it is interesting to note that H9 is the least stable helix in those cases (Figure 5) since it belongs structurally to the H9–H12 bundle. Unraveling of H9 is the first major unfolding event of H1–H9 even if the force is distributed over terminal helices, since H9 is easily detached from the H1–H8 bundle.

### The Vinculin Binding Sites (VBS) Are Sequentially Exposed once the Talin Rod Breaks Up

Activation of the VBS requires that the VB helices H4, H6, H9, H11, and H12, are at least partially exposed to water. As seen in Figure 3D, these five VB helices are buried in the H1–H12 bundle under equilibrium, and become sequentially exposed only after the fragmentation of the talin rod into smaller helix bundles has occurred. The asterix defines the point where the strain-exposed solvent-buried surface area of a VB helix is equal to the solvent-buried area of the helix when complexed to the vinculin head. The asterix thus marks the unique points in the unfolding trajectory in which each of the VB helices gets activated (Figure 3D). Breaking the talin rod apart thus defines the highest energy barrier that has to be overcome to initiate the exposure and activation of the VBSs, and the implications thereof will be discussed below.

### Hydrogen Bonding Analysis of the Interfaces between Helix Bundles

To better understand the molecular mechanism behind the interactions that regulate helix bundle separation, we analyzed the hydrogen bonding pattern between the defined helix bundles as shown in Figure 4. The blue fluctuatuions represent bonds formed between H1–H5 and H6–H8, while the green represents bonds formed between H6–H8 and H9–H12. While most hydrogen bonds fluctuate between formation and breakage even during the equilibration, only a few of the side-chain hydrogen bonds, like the bond 2, are longer-lived. This, together with the fact that there appears to be considerable statistical variability in bond breaking events between different simulations suggests that at least most of these side-chain hydrogen bonds are not force-bearing. While it cannot be excluded that one or the other of these bonds slightly contributes to the mechanical stabilization, hydrophobic contacts between the helix bundles seem to play the dominant role in upregulating the mechanical stability of the N-terminal part of the talin rod.

A separate SMD analysis of the talin rod fragments H1–H5 was done recently by applying force to the polar side chains T498, S501 and S502 close to the N-terminus of H1 and Q635, Q646, E650, and Q653 close to the C-terminus of H5, assuming that the force-bearing interactions were mediated by side chain hydrogen bond formation across the interfaces of adjacent α-helix bundles [41]. When using an implicit water model in which the protein structure was solvated in a dielectric medium, they observed a rotation of VBS1 (H4) under applied force and suggested this to be a potential activating mechanism. They also observed that the H4 rotation was strongly reduced when repeating the simulation in the presence of explicit water molecules (these computationally more elaborate conditions were used in our simulations as well). We thus analyzed for how long the polar side chains of H5 are hydrogen-bonded across the interface formed between H1–H5 and H6–H8. Our simulations of H1–H12 reveal that water penetrates into the interface H1–H5 and H6–H8, thus breaking these side-chain hydrogen bonds, even before we can see a major force-induced structural change within the H1–H5 bundle (Figure S3). Among the residues that were previously used to model the contact interface between bundles H1–H5 and H6–H8 [41], only residues Gln635 (bonded to Ser714) and Gln646 (bonded to Lys721) are hydrogen bonded across the interface, and those side-chain bonds show quite low stability during our simulations (Figures 4, bonds 6 and 14, and S3), suggesting that those polar residues are also not force-bearing during the activation process of the H4 helix.

### Comparative Analysis of Talin1 and Talin2

To compare the mechanical properties of the talin1 and talin2 rods, H1–H12, we generated a homology model of talin2 based on the talin1 structure (Figure S4). Homology modeling is a reasonable approach since their sequences are highly similar (74% identical) in this region. Furthermore, a sequence analysis of the hydrogen bonding partners when comparing talin1 and talin2 from human, mouse, and chicken (Figure 4) revealed that 29 of the 43 residues participating in hydrogen bonds between helix bundles are fully conserved, eleven of the residues (Ser658, Ser688, Thr693, Thr720, Ser754, Gln755, Gln762, Arg765, Glu780, His788, Lys869) are similar and only three residues are non-conserved (Arg692, Ser752, Gly766) (Figure S1). The RMSD for backbone atoms after energy minimization and thermalization was 1.3 Å. Also for talin 2, the fragmentation of the rod H1–H12 constituted the major energy barrier as seen in constant force and constant velocity SMD simulations, in which the force was distributed over force-bearing interfaces. The split of the rod occurred in identical positions as described above for talin1, namely between the bundles H1–H5, H6–H8 and H9–H12. Also the sequence of early events was similar: First, the H9–H12 bundles separated, followed by the rupture of the H1–H8 fragment. Our simulations of early events further indicate that the major energy barrier of fragmenting the rod are not greatly different between the talin1 and talin2 rods, H1–H12, at least within the stochastic variability that is intrinsic to single molecule studies (Figure S5).

### Forces and Timescale of Computer Simulations

Finally, it is important to note that the SMD simulations are carried out on time scales that differ significantly from those at which biological molecules are stressed. Since the force needed to unfold a protein is logarithmically dependent on the pulling velocity, significantly smaller forces may be able to cause the here described structural rearrangements at physiological timescales. Unfolding forces measured by SMD in nanosecond timescale are thus significantly higher compared to those measured using AFM on millisecond timescales, yet, SMD has correctly predicted in the past the relative mechanical stabilities of some protein domains and the position of key energy barriers [32–34].

## Discussion

Building on the previous experimental demonstration that the VBS are biologically inactive in the intact talin rod while they are activated in isolated bundle fragments [30], we present a first structural model here of how mechanical force can break the talin rod, H1–H12, into smaller α-helix sub-bundles, namely H1–H5, H6–H8 and H9–H12 (Figures 2, 3, and 5). The mechanical stability of the intact talin rod differs significantly whether force is applied to the terminal atoms (Figure 2), or if it is distributed over two force-bearing interfaces that the rod fragment H1–H12 might form with the talin head and the rest of the talin rod (Figure 3), respectively. Tight packing of helix bundles might thus prevent the gradual unraveling of terminal helices (Figure 3) as also observed for other proteins [42]. In contrast, force applied to just the terminal atoms induces unraveling of the terminal helices, turn-by-turn, and reduces the force needed to disintegrate the remaining bundle (Figure 2). Consequently, the two vinculin binding helices H11 and H12 lose their secondary structure early in the unfolding trajectory and might be deactivated by stretching them into straightened polypeptide chains. Fragmentation of the rod into smaller helix bundles is the major structural event in the forced-unfolding pathway and initiates the force-induced activation (asterisk in Figure 3D) of the vinculin binding helices, H6, H9, H11, and H12 (Figures 3 and 5; Table 1). Each of these VB helices has a unique activation point while the VB helix H4 remains buried in the H1–H5 bundle until this bundle disintegrates at later times (Figure 5). All simulations have shown the same fracture planes between the bundles, even when comparing the rod segments of talin1 and talin2.

**Table 1:**
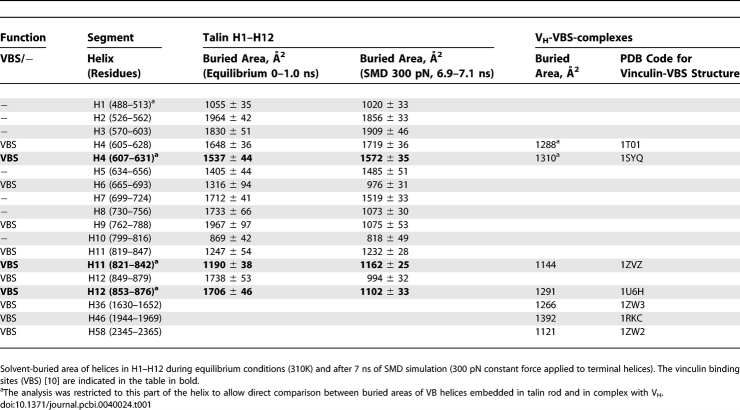
Buried Surface Areas of VB Helices in the Talin Rod and in Complex with the Vinculin Head

Distributing the force across force-bearing interfaces is thus probably a better physiological representation of how force might be transmitted through the intact talin rod. Our simulations further suggest that the H1–H12 bundle is mainly held together via hydrophobic interactions (Figure 4), and that the mechanically more labile smaller α-helix bundles H1–H5, H6–H8, and H9–H12 are stabilized if the bundles are packed closely against each other (Figures 2 and 5). Our findings for the talin rod are consistent with simulations of the mechanical properties of other proteins. Also ankyrin which has a rod composed of densely packed helices tilted away from its long axis show that the initial rod fragmentation is associated with the highest energy barrier [43]. In contrast, for proteins where the helices align with the long axis of the molecule, for example for spectrin repeats, it has been observed that they unfold at relatively low forces [44].

Once water penetrates into the strained talin rod, the putative fracture planes loosen up, thereby allowing single water molecules to slip in, and finally open up the buried interfaces between adjacent helices (Figure 3). This notion of a force-induced water exposure of the VB helices, once the talin rod has fragmented into smaller pieces, is consistent with experimental findings that the VBS can be exposed under equilibrium conditions, but only if point mutations destabilize the integrity of the talin rod [27]. Identifying the planes along which the talin rod fragments if the rod is mechanically strained is important, since mechanical and chemical unfolding pathways can be significantly different [31,45].

### Structural Insights into How Talin's Vinculin Binding Sites Are Sequentially Exposed by Force

Five VB helices have been identified in H1–H12 (Figures 1 and S1; Table 1) [10], namely H4, H6, H9, H11, and H12. Breaking the H1–H12 talin rod into smaller bundles is the prerequisite for the strain-dependent decrease in the buried surface area of these amphiphatic VB helices (Figure 3). Starting with the first split where H1–H8 separates from the H9–H12 bundle (Figure 3), we observe a significant decrease in the buried surface areas of the three VB helices (H6, H9, and H12), while the buried surface area of the VB helix H4 is decreasing only at later time points when helix bundle H1–H5 is fragmented into smaller pieces. Note that H6, H9 and H12 are located at the interfaces of the H1–H5, H6–H8 and H9–H12 bundles. Since H11 is buried in the interior of the subbundle, there is only a slight drop in the buried area observed during the simulated time frame.

Is the increased solvent exposure sufficient to activate the VBSs? Considerable experimental evidence has demonstrated that bundle fragmentation by clipping off helices can indeed expose the VB sites that are otherwise buried in the full length talin rod under equilibrium conditions. The isolated H1–H12 bundle shows a higher affinity to vinculin than does the full-length talin or talin rod domain [30], and mutations stabilizing the H9–H12 bundle result in decreased vinculin binding [30]. NMR studies have revealed that the helix H10 of the isolated H9–H12 bundle unfolds into a flexible random coil when the fragment complexes with V_H_ [28]. Taken together, these results support the model where the H9–H12 bundle is stabilized by the neighboring helices which bury the VB helices, H9, H11 and H12. Similar trends are seen for the VB helix H4. Experiments show that the H1–H5 bundle does not bind to vinculin but removal of H5 from the bundle activates the otherwise buried VB helix H4 [30].

### A Structural and Energetic Model of How Force Might Activate Talin Binding to Vinculin

Taking together all of these experimental and computational findings, we propose the following model of how tensile force acting on talin can activate its binding to vinculin:

#### Equilibrium.

The helix bundles of the talin rod are tightly packed against each other and stabilize each other mechanically as demonstrated here by SMD. The amphiphatic VB helices are buried as seen in Figure 3 (see also [27,28]) and thus deactivated, and their buried areas are larger than if complexed with vinculin (Table 1).

#### Force-induced fragmentation of the talin rod, H1–H12.

Tensile force applied to the talin rod promotes its fragmentation into smaller α-helix bundles. Breaking the rod constitutes the major first event but ***only*** if the tensile force is applied via force-bearing interfaces but not if the force is transmitted only via the terminal atoms (as typically done in single molecule force pulling experiments) (see Figures 2 and 3). Assuming that the force is applied to the intact talin rod via force-bearing interfaces is most likely the physiologically more relevant scenario (see Results).

#### Gradual exposure of VB helices to water.

Once water starts to penetrate into a bundle-bundle interface, the neighboring bundles separate which leads to an increased water exposure of VB helices. Out of the five VB helices, only H6, H9 and H12 directly border the bundle-bundle fracture planes identified in this study (Figure 3), and our simulations predict that they are the first once to get activated.

#### Point at which a VB helix is fully activated (see asterisk in Figure 3D).

**Figure 6:**
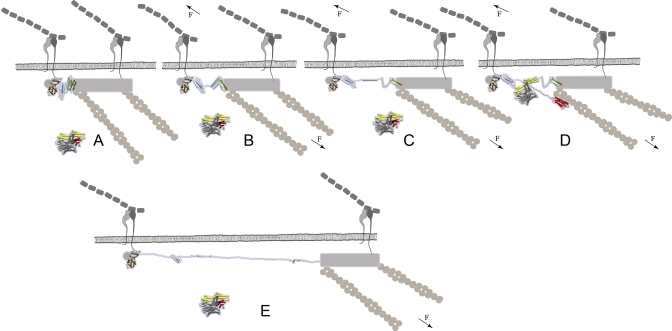
Cartoon Model Shows How Vinculin Binding to Talin Might Be Force-Activated VB helices are presented as colored cartoons, and other helices in H1–H12 (PDB 1XWX) are shown in light gray cartoon models. The gray shaded areas represent the talin regions for which no high resolution structures are available. The vinculin cartoon is based on the X-ray structure of autoinhibited full length vinculin (PDB 1TR2). (A) No force applied: When talin is not stressed, vinculin has low affinity for talin. Talin forms a force-bearing linkage between the extracellular matrix (gray) bound integrins and actin filaments (shown in brown). It remains unclear whether talin is parallel or tilted with respect to the cell membrane, and whether it forms a dimer. (B) Prerequisite for force-activation: When mechanical force is applied to the integrin-actin linkage, talin is stretched. Force causes the breakage of the talin rod into helix sub-bundles, which constitutes the major energy barrier. The subbundles H1–H5, H6–H8, and H9–H12 differ in their mechanical stabilities leading to a hierarchal sequence in which they break apart. The forced unfolding pathway of the talin rod might be altered by interaction with other molecules, including PIP2 and vinculin [30], or the potential the dimerization of talin. (C) Activation of talin's VB helices: Continued talin extension causes sequential exposure of the VB helices to water and leads finally to a separation from their host bundles. While the buried surface area of the VB helices in unstrained talin is larger than if comlexed with vinculin, conformational strain gradually exposes their hydrophobic residues—once activated, they can form an energetically more favored complex with the unstrained vinculin head. In this schematic model, only one vinculin molecule is shown. Since there are multiple vinculin binding sites in talin, the number of exposed VB helices is dependent on the applied force and the force-exposure time of talin. (D) Vinculin binding to talin is enabled by α-helix swapping: a water-exposed VB helix can minimize its free energy by associating with the α-helix bundle of the vinculin head thereby burying its VBSs from water. Experiments indicate that the interaction of the vinculin head with a talin VB helix releases the V_H_–V_T_ interaction [22–26] and thus allows the V_T_ (V_T_ domain shown in red) to bind to actin. The linker connecting V_T_ to the rest of the vinculin is constructed manually. (E) Prolonged exposure to tensile mechanical force may cause complete unraveling of individual VB helices, and we propose that this leads to a deactivation of vinculin binding. However, binding of vinculin to a VH helix might stabilize the VB helix from force-induced unfolding.

Once water penetration into the fracture planes is initiated, the buried surface area of a VB helix within the talin rod decreases gradually, not abruptly, under the influence of the external force. The force-induced reduction in buried surface area has an energetic price: the free energy of the system increases proportional to the increase of the water accessible hydrophobic surface area of the VB helices. When an adequate increase in free energy is reached, the partially water-exposed, ‘activated' helix can thus spontaneously associate with the α-helix bundle of the vinculin head (Figure 6) for thermodynamic reasons discussed below. Once activated, a VB helix can associate with the V_H1_ domain of vinculin upon collision such that its hydrophobic side faces the α1 and α2 helices of the vinculin head [10,27–29] (Figure 6). The speed by which this occurs thus depends on the collision rate and thus on the vinculin concentration.

#### Vinculin binding (on-rate) is biphasic since complete unraveling of a VB helix into a stretched polypeptide might ultimately eliminate vinculin binding.

At later time-points of the force-induced unfolding trajectories, it can be seen that the VB helices sequentially unravel completely and are kept in a straightened conformation by the externally applied tensile force. Since refolding of the secondary helix structure against a tensile force would be required to have a VB helix associate with the vinculin head, we propose that force-induced unfolding of a VB helix into a stretched polypeptide chain ultimately inhibits vinculin binding.

This proposed deactivation mechanism is supported by previous force-spectroscopy experiments conducted on other proteins showing that spontaneous refolding is significantly slowed down by tensile force [46]. It should be noted though that vinculin, once bound to a VB helix, might stabilize it against force-induced unraveling, however, such simulations go beyond the scope of this paper.

#### The talin rod as a force-time integrator.

Since the mechanical stabilities of the host bundles of the VB helices vary, and since some VB helices are found in the center of the bundles (H4 and H11) whereas others are located at bundle-bundle interfaces (H6, H9, H12), we see that each helix becomes hydrated at a different time point in the unfolding trajectory (Figure 3D). Accordingly, we expect that each VB helix has its distinct biphasic activation and deactivation response which is force and time dependent.

### An α-Helix Swap Mechanism of How Force Might Accelerate the Bond Formation Rate of Vinculin to Talin

What drives the force-induced association of talin helices with vinculin once they have been activated by water exposure? Key to the VB helices are hydrophobic residues that are located on one face of these amphiphatic α-helices which share the consensus (LxxAAxxVAxxVxxLIxxA), where x is a variable amino acid residue [10]. As shown for talin's isolated VB helices H4, H11 and H12 (the only ones for which sufficient data are available), association with the vinculin head is energetically favored since the vinculin head is thermodynamically stabilized by complexation with VB helices [23,26], and has considerable structural homology to the respective equilibrium talin bundle structures [10,27,28]. At equilibrium, the buried surface areas of talin's VB helices complexed with talin versus the *vinculin head*, respectively, are 1537 Å^2^ *vs. 1310* Å^2^ for H4 (residues 607–631), 1190 Å^2^ versus *1144* Å^2^ for H11 (residues 821–842), and 1706 Å^2^ versus *1291* Å^2^ (residues 853–876) (Table 1). Accordingly, the affinities of isolated VB helices with vinculin (K_d_ = 3–33 nM) [47] differ considerably from that of full length talin (K_d_ = 8.9 μM) [30]. It has thus been proposed earlier that once released from the hydrophibic core of the talin rod, the H4 (VBS1) and potentially the other VB helices are available to induce ‘bundle conversion' thereby displacing the intramolecular interaction of the vinculin head to its tail [24,27,28,48]. This suggestion was further supported by findings that vinculin can be activated by helical peptides that contain the VBSs, subsequently leading to an increased affinity of the vinculin tail to actin [22, 23].

Following these earlier suggestions derived for unstrained systems, we now propose that the complementary design between the talin and vinculin structures facilitates ***force-induced*** α-helix swapping: the VB helices find a thermodynamically more stable environment in talin *under equilibrium* conditions (Table 1), the biologically inactive state, but as soon as talin is sufficiently strained, the association with the unstrained vinculin head is energetically preferred once a collision has occurred and occurs upon a collision with the vinculin head. Vice versa, the vinculin head is thermodynamically less stable in the absence of a swapped talin helix [30] and thus forms the auto-inhibitory complex with its tail [23–26]. We further suggest a biphasic vinculin binding behavior: the maximum probability of the α-helix swapping is reached once the VB helix has broken away from the other talin helices and exposes the hydrophobic residues of the otherwise structurally intact VB helices to water (Figure 6). Once a VB helix is sufficiently stretched and starts to lose its secondary structure, helix swapping might again be inhibited.

Of major physiological importance is furthermore the insight that the talin rod is engineered such that the activation of vinculin binding is force *and* time dependent, thus acting as a hierarchical force-time integrator. Not all VB helices are activated at the same time since the VB helices are located in sub-bundles that break up sequentially due to their differential mechanical stabilities (Figures 3 and 5). A hierarchy thus exists in which the VBSs are force-activated (as indicated by asterixes in Figure 3D): the earliest to be exposed are the VB helices H6 and H12, which are located in the weakest helix bundle interface. The next to be exposed are the VB helices H9 and H11 located in the C-terminal H9–H12 bundle, and the last one is VB helix H4, which is located in the sub-bundle H1–H5. If the talin rod is kept under constant force, the number of VB helices that have been activated will initially increase with time and the amount of force applied. Once a vinculin complex has been formed during the lifetime of the activated helix, the complexed helix might be protected against complete unraveling due to the additional interactions formed between a VBH and the unstrained vinculin head. In contrast, when applying force to the terminal ends of H1–H12, the VB helices H11 and H12 lose already their secondary structure before the bundle is completely fractured, potentially leading to their early deactivation.

Vinculin recruitment to talin thus initially increases if talin is incorporated into a force-bearing network formed when a cell adheres to a surface or matrix fibrils [15–18,20,29,30]. However, since each VB helix has its unique biphasic time response, increased force thus accelerates the sequence of these events, but not necessarily the total number of activated VBSs at later time points, particularly at low vinculin concentrations.

The comparative analysis carried out here for the rod fragment H1–H12 of talin1 and talin2 shows that they share a similar sequence of early unfolding events that lead to the fragmentation of the N-terminal part of the talin rod. Talin1 and talin2 are not only differentially expressed in various tissues but also localize in different parts of a cell [49]. While we do not see major changes between the mechanical stabilities of the H1–H12 fragments of two talins, i.e., in their likelihood of being fragmented by tensile force, it is important to note that the talin rod has six additional VBSs in the structurally unresolved C-terminus and that we do not have any information so far regarding the differential mechanical stresses to activate them (see Figures 1B and S1). Unclear is also to what extend functional differences of talin1 and talin2 might result from mutations in their binding sites to other proteins.

### Repertoire of Force-Activated Bonds: A More General Mechanism?

The mechanism described here might not be unique to the talin-vinculin bond but might be more widespread among proteins that are composed of α-helical bundles. First of all, once an amphiphatic helix is broken off from a helix bundle by the application of tensile force, it might be stabilized by insertion into either hydrophobic pockets of other proteins or even into the lipid bilayer [27]. Alternatively, other proteins that form helix bundles might also bind vinculin in a force-regulated manner. α-actinin, for example, has also a VB helix that can form a similar structural complex with vinculin [23,26]. Similarly to talin, the VBS in α-actinin is buried in the native structure [50]. Identifying the repertoire of mechanisms by which forces can upregulate adhesive interactions has led to the recent discovery of catch bonds where a receptor-ligand interaction is enhanced when tensile mechanical force is applied between a receptor and its ligand (for review see [51,52]). In contrast, the force-activated helix swapping mechanism proposed here requires that the force is applied to just one of the binding partners, thereby activating bond formation with a free ligand. Also in contrast to catch bonds, the ligand does not necessarily have to be part of the force-bearing protein network at the time the swap is initiated. While force-induced helix swapping thus primarily upregulates the bond formation rate, the catch bond mechanism primarily extends the lifetime of an already existing complex.

## Materials and Methods

### Starting structures.

The talin rod domain H1–H12 (residues 486–889) of mouse talin was obtained from the Protein Data Bank (http://www.pdb.org/ [PDB theoretical models section: 1XWX]) [28]. This structure is an energy-minimized model derived from combining two talin fragments derived from NMR (H9–H12, residues 755–889 [PDB: 1U89] [28]) and X-ray (H1–H9, residues 482–789 [PDB: 1SJ8] [27]). Another talin structure was also studied (H1–H9 [PDB: 1SJ8]). Talin fragments H2–H8 (residues 523–757) and H9–H12 (residues 755–882) were obtained from the 1XWX talin rod structure by removing the excess atoms.

Homology model for talin2 H1–H12 was generated based on talin1 [PDB: 1XWX] using SWISS-MODEL protein structure homology-modeling server [53] and subjected to WhatCheck analysis. The human talin2 sequence was used; human and mouse talin2 are virtually identical in the region of H1–H12, and there are only 2 sequence differences: the residue corresponding to human talin2 Glu604 is Asp in mouse, and the residue corresponding human to talin2 Thr762 is serine in mouse talin2. The sequence alignment was prepared using ClustalW (http://www.ebi.ac.uk/clustalw/). The H1–H12 sequences of talin1 and talin2 have 74% identity.

### Molecular dynamics.

All the simulations were carried out in explicit water. The periodical TIP3 water box was created using the program VMD plugin solvate [54] The system was neutralized by adding Na^+^- or Cl^−^-ions to the system using VMD autoionize-plugin. The systems used in the simulations are summarized in Table S1.

Since efficient arrangement of water is a pre-exquisite for validity of the results, we used Solvate 1.0 (http://www.mpibpc.mpg.de/groups/grubmueller/start/software/solvate/docu.html) to find possible hydration sites inside the protein, and energetic evaluation of those positions was performed using Dowser [55]. Positions energetically favoring hydration were analyzed in the H1–H12 which was subjected to a 1 ns equilibration after solvation with the VMD solvate plugin. A further analysis revealed that 1 ns equilibrations were sufficiently long to allow water to penetrate into all energetically favored locations.

A 12 Å cutoff was used for the van der Waals interactions, switching function starting from 10 Å. The PME (Particle Mesh Ewald) method was used to calculate long-range electrostatics without a cutoff using grid spacing lower than 1/Å^3^.

Each system was minimized with a conjugate gradient method using NAMD [56]. Initially, only the solvent and ions were allowed to move for 4,000 steps. Next, the entire system was allowed to move for another 4,000 steps. After minimization, the system was heated from 0 K to 300 or 310 K in 30 ps under Berendsen pressure control (1 atm) and subsequently equilibrated for 1 ns under constant pressure (Berendsen pressure control at 1 atm) and temperature (tCouple method). Structures equilibrated for 1 ns were used in all SMD simulations.

### Steered molecular dynamics.

Constant force was applied either to both termini, or alternatively to C_α_-atoms of the terminal helices (residues 495–514 and 853–872) and the force vector was then calculated using residues in the middle of the helices (residues 504 and 865). The system coordinates were saved every picosecond, and the system energies were recorded every 0.1 ps.

Constant velocity SMD simulations were performed by applying moving springs with a spring constant of 5 kcal/mol/Å^2^ [30] to the C_α_-atoms of residues 853–872 in the C-terminal helix H12. The springs were moved along the force vector calculated according to the vector connecting the C_α_-atoms of residues 504 and 865 with a velocity of 1, 10, or 100 Å/ns, while the C_α_-atoms of the residues 495–514 of the N-terminal helix H1 were constrained using a harmonic energy constraint function with a spring constant of 5 kcal/mol/Å^2^. The force along the pulling vector was obtained from the absolute forces measured during the simulation by calculating the projection of the force vector to the pulling vector.

Pressure was maintained at 1 atm by the Berendsen pressure control method implemented in NAMD with the following parameters: BerendsenPressureCompressibility 0.0000457, BerendsenPressureRelaxationTime 1000, BerendsenPressureFreq 4. The temperature of the system was maintained at a defined temperature using the tCouple method implemented in NAMD with a tCouple coefficient of 1.

### Analysis.

The resultant trajectories were analyzed using VMD version 1.8.5 [54]. The pictures were rendered using Tachyon rendering system implemented in VMD and further processed with programs GIMP and Adobe Photoshop CS2.

Surface-accessible surface area (SASA) was measured using VMD with a 1.4 Å scanning probe. The vinculin head complexed to talin VBSs was first hydrogenated using the program psfgen. The structures were then subjected to surface analysis without further processing. The analysis was done for both the whole protein and individual helices. Calculations over simulation trajectories were performed for 10 ps spacing between frames. The buried areas of VB helices were calculated according to Eq. 1.

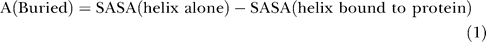



To avoid the burying effect of the backbone of the neighboring talin polypeptide chain, one residue before and after each helix was excluded from the analysis. This makes direct comparisons to vinculin–VBS complexes more accurate, since VBS-helices are individual peptides in these complexes and therefore not connected to a protein scaffold as are helices in the talin rod.

### Sequence analysis of talins.

Sequence alignments for human, mouse and chicken talin1 and talin2 proteins were done using program ClustalW using default parameters. The RefSeq database (http://www.ncbi.nlm.nih.gov/RefSeq/index.html) accession numbers used are as follows: human talin1 (NP_006280); human talin2 (NP_055874); mouse talin1 (NP_035732); mouse talin2 (XP_486227); chicken talin1 (NM_204523). The sequence of chicken talin2 was combined from database sequences (XP_413760) and (XP_413761).

### Computation.

Computation was carried out on the Gonzales cluster at ETH Zürich (2.4 GHz AMD Opteron 250 processors) and at CSCS Cray XT3 (2.6 GHz AMD Opteron processors). For 1 ns of simulation time (∼100,000 atoms), ∼1100 cpu hours were needed on both clusters. Overall, >200 ns of simulation data were analyzed in this study.

## Supporting Information

Figure S1 Sequence Analysis of Talin1 and Talin2 from Human, Mouse, and Chicken Secondary structure of the talin head region is shown according to (PDB: 1MK7). Secondary structure for the first 200 residues is missing. The secondary structure in the talin rod region (residues 400-2541) is shown according to the previously published predicted structure [10]. The unknown sequence in chicken talin2 (residues 173–283) was not taken into account when the consensus shown below the alignment was calculated using ClustalW. This unknown region is also excluded from the sequence numbering of chicken talin2. RefSeq accession numbers are: human talin1 (NP_006280); human talin2 (NP_055874); mouse talin1 (NP_035732); mouse talin2 (XP_486227); chicken talin1 (NM_204523). The sequence of chicken talin2 was combined from database sequences (XP_413760) and (XP_413761). The calpain cleavage site between the talin head and rod domains is shown with a pink background (cleavage site is in the middle of the LQQQ sequence). The secondary structure is shown above the alignment: arrow, beta-strand; H, helix. Helices corresponding to vinculin binding sites are labelled by yellow shading in secondary structure indicator [10] and the VBSs in the talin H1–H12 fragment are indicated by color shading in the helix name.
(91 KB PDF)

Figure S2 Constant Velocity SMD Analysis for Talin1 and Talin2 Fragment H1–H12 Constant velocity SMD simulations were carried out by moving springs attached to helix H12 with a velocity of 10 Å/ns, while the H1 was fixed by application of harmonic constrains. The force needed to move the springs is plotted in the figure over the position of the atoms where the springs were attached, and structural snapshots are shown for talin1 (A) and talin2 (B). To give a view of the landscape of the force, a cubic spline was fitted to the data (black line). The positions of the intermediate states found in constant force simulations for talin1 (Figure 3) are shaded in (A).
(7.3 MB TIF)

Figure S3 Location of the Residues Where Others Applied Force in the Interface between Bundles H1–H5 and H6–H8 The residues where the force is applied in the study of Lee et al. [41] are shown in stick representation. The structures of talin1 H1–H12 after a 1 ns equilibration and after a 5.5 ns SMD simulation (300 pN applied distributed along the terminal helices) are shown.
(6.0 MB TIF)

Figure S4 Superposition of the Talin1 and Talin2 Structures, H1–H12 The structures are superimposed after a 1 ns equilibration. The structural alignment omitted the C-terminal flexible tail. Talin1 and talin2 are shown in colorful cartoon and gray cartoon representations, respectively. The side chains of non-conserved residues are shown in sticks (talin1 yellow, talin2 gray).
(3.5 MB TIF)

Figure S5 Constant Force SMD Simulation for Talin2 Constant force simulations were carried out by applying constant force, which was distributed over the terminal helices. The distance between the Cα-atoms of residues 504 and 865 was followed during the simulation (residue numbering according to talin1). Two simulations are shown in the case of 300 pN and 400 pN.
(385 KB TIF)

Table S1 Protein Fragments Used in This Study (32 KB DOC)

## Abbreviations

FAK: focal adhesion kinase
SMD: steered molecular dynamics
T_H_: talin head
T_R_: talin rod
VBS: vinculin binding site, V_H_, vinculin head
V_T_: vinculin tail
