# Supplementary material for: How Force Might Activate Talin's Vinculin Binding Sites: SMD Reveals a Structural Mechanism
Source: PLoS Comput Biol. 2008 Feb 15;4(2):e24. doi: 10.1371/journal.pcbi.0040024 (PMC2242828; doi:10.1371/journal.pcbi.0040024)
Supplement: Supplementary file 1 [file pcbi.0040024.sg001.pdf]

Fig. S1

```
Human talin1     MVALSLKISIG--NVVKTMQFEPSTMVYDACRIIRERIPEAPAGPPSDFGLFLSDDDPKK 58
Mouse talin1     MVALSLKISIG--NVVKTMQFEPSTMVYDACRMIRERIPEALAGPPNDFGLFLSDDDPKK 58
Chicken talin1   MVALSLKISIG--NVVKTMQFEPSTMVYDACRMIRERVPEAQMGQPNDFGLFLSDEDPKK 58
Human talin2     MVALSLKICVRHCNVVKTMQFEPSTAVYDACRVIRERVPEAQTGQASDYGLFLSDEDPRK 60
Mouse talin2     MVALSLKICVRHCNVVKTMQFEPSTAVYDACRVIRERVPEAQTGQASDYGLFLSDEDPRK 60
Chicken talin2   MVALSLKICVRQCNVVKTMQFEPSTAVYDACRVIRERVPEAQMGQASDYGLFLSDEDPRK 60
                 ********.:   ************ ******:****:***  * ..*:******:**:*

Human talin1     GIWLEAGKALDYYMLRNGDTMEYRKKQRPLKIRMLDGTVKTIMVDDSKTVTDMLMTICAR 118
Mouse talin1     GIWLEAGKALDYYMLRNGDTMEYRKKQRPLKIRMLDGTVKTIMVDDSKTVTDMLMTICAR 118
Chicken talin1   GIWLEAGKALDYYMLRNGDTMEYKKKQRPLKIRMLDGTVKTVMVDDSKTVTDMLTTICAR 118
Human talin2     GIWLEAGRTLDYYMLRNGDILEYKKKQRPQKIRMLDGSVKTVMVDDSKTVGELLVTICSR 120
Mouse talin2     GIWLEAGRTLDYYMLRNGDILEYKKKQRPQKIRMLDGSVKTVMVDDSKTVGELLVTICSR 120
Chicken talin2   GIWLEAGRTLDYYMLRNGDVLEYKKKQRPQKIRMLDGSVKTVMVDDSKTVGELLVTICSR 120
                 *******::********** :**:***** *******:***:******** ::* ***:*

Human talin1     IGITNHDEYSLVRELMEEKKEEITGTLRKDKTLLRDEKKMEKLKQKLHTDDELNWLDHGR 178
Mouse talin1     IGITNHDEYSLVRELMEEKKDEGTGTLRKDKTLLRDEKKMEKLKQKLHTDDELNWLDHGR 178
Chicken talin1   IGITNYDEYSLVREIMEEKKEEVTGTLKKDKTLLRDEKKMEKLKQKLHTDDELNWLDHGR 178
Human talin2     IGITNYEEYSLIQETIEEKKEEGTGTLKKDRTLLRDERKMEKLKAKLHTDDDLNWLDHSR 180
Mouse talin2     IGITNYEEYSLIQETIEEKKEEGTGTLKKDRTLLRDERKMEKLKAKLHTDDDLNWLDHSR 180
Chicken talin2   IGITNYEEYSLIQESIEEKKEESTGTLKKDRTLLRDERKMEKLKAKLHTDDDC------- 180
                 *****::****::* :****:* ****:**:******:****** ******: *****.*

                                               HHHHHHHHHHHHHHHHH     HHHHHHHH
Human talin1     TLREQGVEEHETLLLRRKFFYSDQNVDSRDPVQLNLLYVQARDDILNGSHPVSFDKACEF 238
Mouse talin1     TLREQGVEEHETLLLRRKFFYSDQNVDSRDPVQLNLLYVQARDDILNGSHPVSFDKACEF 238
Chicken talin1   TLREQGIDDNETLLLRRKFFYSDQNVDSRDPVQLNLLYVQARDDILNGSHPVSFDKACEF 238
Human talin2     TFREQGVDENETLLLRRKFFYSDQNVDSRDPVQLNLLYVQARDDILNGSHPVSFEKACEF 240
Mouse talin2     TFREQGVDENETLLLRRKFFYSDQNVDSRDPVQLNLLYVQARDDILNGSHPVSFEKACEF 240
Chicken talin2   ------------------------------------------------------------
                 *:****::::********************************************:*****

                 HHHHHHHHHH            HHHH  HHHHHHH H HHHHHHHHHH    HHHHHHHH
Human talin1     AGFQCQIQFGPHNEQKHKAGFLDLKDFLPKEYVKQKG-ERKIFQAHKNCGQMSEIEAKVR 297
Mouse talin1     AGFQCQIQFGPHNEQKHKAGFLDLKDFLPKEYVKQKG-ERKIFQAHKNCGQMSEIEAKVR 297
Chicken talin1   AGYQCQIQFGPHNEQKHKPGFLELKDFLPKEYIKQKG-ERKIFMAHKNCGNMSEIEAKVR 297
Human talin2     GGFQAQIQFGPHVEHKHKPGFLDLKEFLPKEYIKQRGAEKRIFQAHKNCGEMSEIEAKVK 300
Mouse talin2     GGFQAQIQFGPHVEHKHKPGFLDLKEFLPKEYIKQRGAEKRIFQEHKNCGEMSEIEAKVK 300
Chicken talin2   -------------------------------------------EEHKNCGEMTEIEAKVK 300
                 .*:*.******* *:***.***:**:******:**:* *::**  *****:*:******:

                 HHHHHHH                                                 HHH
Human talin1     YVKLARSLKTYGVSFFLVKEKMKGKNKLVPRLLGITKECVMRVDEKTKEVIQEWNLTNIK 357
Mouse talin1     YVKLARSLKTYGVSFFLVKEKMKGKNKLVPRLLGITKECVMRVDEKTKEVIQEWSLTNIK 357
Chicken talin1   YVKLARSLKTYGVSFFLVKEKMKGKNKLVPRLLGITKECVMRVDEKTKEVIQEWSLTNIK 357
Human talin2     YVKLARSLRTYGVSFFLVKEKMKGKNKLVPRLLGITKDSVMRVDEKTKEVLQEWPLTTVK 360
Mouse talin2     YVKLARSLRTYGVSFFLVKEKMKGKNKLVPRLLGITKDSVMRVDEKTKEVLQEWPLTTVK 360
Chicken talin2   YVKLARSLRTYGVSFFLVKEKMKGKNKLVPRLLGVTKDSVMRVDEKTKEVLQEWPLTTVK 360
                 ********:*************************:**:.***********:*** **.:*

                                            HHHHHHHHHHHHHHH
Human talin1     RWAASPKSFTLDFGDYQDGYYSVQTTEGEQIAQLIAGYIDIILKKKKSKDHFGLEGDEES 417
Mouse talin1     RWAASPKSFTLDFGDYQDGYYSVQTTEGEQIAQLIAGYIDIILKKKKSKDHFGLEGDEES 417
Chicken talin1   RWAASPKSFTLDFGDYQDGYYSVQTTEGEQIAQLIAGYIDIILKKKKSKDHFGLEGDEES 417
Human talin2     RWAASPKSFTLDFGEYQESYYSVQTTEGEQISQLIAGYIDIILKKKQSKDRFGLEGDEES 420
Mouse talin2     RWAASPKSFTLDFGEYQESYYSVQTTEGEQISQLIAGYIDIILKKKQSKDRFGLEGDEES 420
Chicken talin2   RWAASPKSFTLDFGEYQESYYSVQTTEGEQISQLIAGYIDIILKKKQSKDRFGLEGDEES 420
                 **************:**:.************:**************:***:*********
```

```
Human talin1     TMLEDSVSPKKSTVLQQQYNRVGKVEHGSVALPAIMRSGASGPENFQVGSMPPAQQQITS 477
Mouse talin1     TMLEDSVSPKKSTVLQQQYNRVGKVEHGSVALPAIMRSGASGPENFQVGSMPPAQQQITS 477
Chicken talin1   TMLEDSVSPKKSTVLQQQFNRVGKAELGSVALPAIMRTGAGGPENFQVGTMPQAQMQITS 477
Human talin2     TMLEESVSPKKSTILQQQFNRTGKAEHGSVALPAVMRSGSSGPETFNVGSMPSPQQQVMV 480
Mouse talin2     TMLEESVSPKKSTILQQQFNRTGKAEHGSVALPAVMRSGSSGPETFNVGSMPSPQQQVMV 480
Chicken talin2   TMLEESVSPKKSTILQQQFNRAGKVEHGSVALPAVMRSGSSGPETFNVGIMPSPQQQVTI 480
                 ****:********:****:**.**.* *******:**:*:.***.*:** ** .* *:
                                      H1
                             HHHHHHHHHHHHHHHHHHHHH             HHHHHHHHHHHHH
Human talin1     GQMHRGHMPPLTSAQQALTGTINSSMQAVQAAQATLDDFDTLPPLGQDAASKAWRKNKMD 537
Mouse talin1     GQMHRGHMPPLTSAQQALTGTINSSMQAVQAAQATLDDFETLPPLGQDAASKAWRKNKMD 537
Chicken talin1   GQMHRGHMPPLTSAQQALTGTINSSMQAVNAAQATLDDFETLPPLGQDAASKAWRKNKMD 537
Human talin2     GQMHRGHMPPLTSAQQALMGTINTSMHAVQQAQDDLSELDSLPPLGQDMASRVWVQNKVD 540
Mouse talin2     GQMHRGHMPPLTSAQQALMGTINTSMHAVQQAQDDLSELDSLPPLGQDMASRVWVQNKVD 540
Chicken talin2   GQMHRGHMPPLTSAQQALMGTINTSMHAVQQAQADLSEVDNLPPLGQDMASRVWVQNKVD 540
                 ****************** ****:**:**: **  *.:.:.******* **:.* :**:*
                        H2                                          H3
                 HHHHHHHHHHHHHHHHHHHHHHH         HHHHHHHHHHHHHHHHHHHHHHHHHHHHH
Human talin1     ESKHEIHSQVDAITAGTASVVNLTAGDPAETDYTAVGCAVTTISSNLTEMSRGVKLLAAL 597
Mouse talin1     ESKHEIHSQVDAITAGTASVVNLTAGDPAETDYTAVGCAVTTISSNLTEMSRGVKLLAAL 597
Chicken talin1   ESKHEIHSQADAITAGTASVVNLTAGDPADTDYTAVGCAVTTISSNLTEMSKGVKLLAAL 597
Human talin2     ESKHEIHSQVDAITAGTASVVNLTAGDPADTDYTAVGCAITTISSNLTEMSKGVKLLAAL 600
Mouse talin2     ESKHEIHSQVDAITAGTASVVNLTAGDPADTDYTAVGCAITTISSNLTEMSKGVKLLAAL 600
Chicken talin2   ESKHEIHSQVDAITAGTASVVNLTAGDPVDTDYTAVGCAITTISSNLTEMSKGVKLLAAL 600
                 *********.*****************.:*********:***********:*******
                                    H4                             H5
                 HH        HHHHHHHHHHHHHHHHHHH        HHHHHHHHHHHHHHHHHHHHH
Human talin1     LEDEGGSGRPLLQAAKGLAGAVSELLRSAQPASAEPRQNLLQAAGNVGQASGELLQQIGE 657
Mouse talin1     LEDEGGNGRPLLQAAKGLAGAVSELLRSAQPASAEPRQNLLQAAGNVGQASGELLQQIGE 657
Chicken talin1   MEDEGGNGRQLLQAAKNLASAVSDLLKTAQPASAEPRQNLLQAAGLVGQTSGELLQQIGE 657
Human talin2     MDDEVGSGEDLLRAARTLAGAVSDLLKAVQPTSGEPRQTVLTAAGSIGQASGDLLRQIGE 660
Mouse talin2     MDDDVGSGEDLLRAARTLAGAVSDLLKAVQPTSGEPRQTVLTAAGSIGQASGDLLRQIGE 660
Chicken talin2   MDDEGGSGEDLLKAARTKGIAVSDLLKA-------PRQTVLTAAGSIGQASGELLRQIGE 653
                 ::*: *.*. **:**:  . ***:**::       ***.:* *** :**:**:**:****
                                     H6                                   H7
                       HHHHHHHHHHHHHHHHHHHHHHHHHHH        HHHHHHHHHHHHHHHHHH
Human talin1     SDTDPHFQDALMQLAKAVASAAAALVLKAKSVAQRTEDSGLQTQVIAAATQCALSTSQLV 717
Mouse talin1     SDTDPHFQDVLMQLANAVASAAAALVLKAKSVAQRTEDSGLQTQVIAAATQCALSTSQLV 717
Chicken talin1   SDTDPRFQDMLMQLAKAVASAAAALVLKAKNVAQKTEDSALQTQVIAAATQCALSTSQLV 717
Human talin2     NETDERFQDVLMSLAKAVANAAAMLVLKAKNVAQVAEDTVLQNRVIAAATQCALSTSQLV 720
Mouse talin2     NETDERFQDVLMSLAKAVANAAAMLVLKAKNVAQVAEDTVLQNRVIAAATQCALSTSQLV 720
Chicken talin2   NETDERFQDVLMSLAKAVANAAAMLVLKAKNVAQVAEDTVLQNRVIAAATQCALSTSQLV 713
                 .:** :*** **.**:***.*** ******.*** :**: **.:****************
                                          H8                            H9
                 HHHHHH       HHHHHHHHHHHHHHHHHHHHHHHHH      HHHHHHHHHHHHHHHH
Human talin1     ACTKVVAPTISSPVCQEQLVEAGRLVAKAVEGCVSASQAATEDGQLLRGVGAAATAVTQA 777
Mouse talin1     ACTKVVAPTISSPVCQEQLVEAGRLVAKAVEGCVSASQAATEDGQLLRGVGAAATAVTQA 777
Chicken talin1   ACTKVVAPTISSPVCQEQLIEAGKLVAKSAEGCVEASKAATNDDQLLKQVGVAATAVTQA 777
Human talin2     ACAKVVSPTISSPVCQEQLIEAGKLVDRSVENCVRACQAATTDSELLKQVSAAASVVSQA 780
Mouse talin2     ACAKVVSPTISSPVCQEQLIEAGKLVDRSVENCVRACQAATSDSELLKQVSAAASVVSQA 780
Chicken talin2   ACAKVVSPTISSPVCQEQLIEAGKLVDRSVENCVRACQAATDDTELLKQVSAAASIVSQA 773
                 **:***:***********:***:** ::.*.** *.:*** * :**: *..**: *:**
                                           H10                       H11
                 HHHHH          HHHHHHHHHHHHHHHHHHHHHHH   HHHHHHHHHHHHHHHHHHH
Human talin1     LNELLQHVKAHATGAGPAGRYDQATDTILTVTENIFSSMGDAGEMVGQARILAQATSDLV 837
Mouse talin1     LNELLQHVKAHATGAGPAGRYDQATDTILTVTENIFSSMGDAGEMVRQARILAQATSDLV 837
Chicken talin1   LNDLLQHIKQHATGGQPIGRYDQATDTILNVTENIFSSMGDAGEMVRQARILAQATSDLV 837
Human talin2     LHDLLQHVRQFASRGEPIGRYDQATDTIMCVTESIFSSMGDAGEMVRQARVLAQATSDLV 840
Mouse talin2     LHDLLQHVRQFASRGEPIGRYDQATDTIMCVTESIFSSMGDAGEMVRQARVLAQATSDLV 840
Chicken talin2   LNDLLQHVRQFASRGEPIGRYDQATDTIMCVTESIFSSMGDAGEMVRQARVLAQATSDLV 833
                 *::****:: .*: . * **********: ***.************ ***:*********
```

```
                                          H12                            H13
                 HHHHHHHH    HHHHHHHHHHHHHHHHHHHHHHHHHHHHH     HHHHHHHHHHHHHH
Human talin1     NAIKADAEGESDLENSRKLLSAAKILADATAKMVEAAKGAAAHPDSEEQQQRLREAAEGL 897
Mouse talin1     NAIKADAEGESDLENSRKLLSAAKILADATAKMVEAAKGAAAHPDSEEQQQRLREAAEGL 897
Chicken talin1   NAIKADAEGETDLENSRKLLSAAKILADATAKMVEAAKGAAAHPDSEEQQQRLREAAEGL 897
Human talin2     NAMRSDAEAEIDMENSKKLLAAAKLLADSTARMVEAAKGAAANPENEDQQQRLREAAEGL 900
Mouse talin2     NAMRSDAEAEIDMENSKKLLAAAKLLADSTARMVEAAKGAAANPENEDQQQRLREAAEGL 900
Chicken talin2   NAMRSDAEAEIDMENSKKLLAAAKLLADSTARMVEAAKGAAANPENEDQQQRLREAAEGL 893
                 **:::***.* *:***:***:***:***:**:**********:*:.*:************
                                              H14
                 HHHHHHHHHHHH     HHHHHHHHHHHHHHHHHHHHHHHHHH        HHHHHHHHH
Human talin1     RMATNAAAQNAIKKKLVQRLEHAAKQAAASATQTIAAAQHAASTPKASAGPQPLLVQSCK 957
Mouse talin1     RMATNAAAQNAIKKKLVQRLEHAAKQAAASATQTIAAAQHAASAPKASAGPQPLLVQSCK 957
Chicken talin1   RMATNAAAQNAIKKKLVHKLEHAAKQAAASATQTIAAAQHAAASNKNPAAQQ-QLVQSCK 956
Human talin2     RVATNAAAQNAIKKKIVNRLEVAAKQAAAAATQTIAASQNAAVSNKNPAAQQ-QLVQSCK 959
Mouse talin2     RVATNAAAQNAIKKKIVNRLEVAAKQAAAAATQTIAASQNAAISNKNPSAQQ-QLVQSCK 959
Chicken talin2   RVATNAAAQNAIKKKIVNRLEIAAKQAAAAATQTIAASQNAAVSNKNTAAHQ-QLVQSCK 952
                 *:*************:*::** *******:*******:*:** : * .:. *  ******
                      H15                             H16
                 HHHHHHHHHHHHHHHH      HHHHHHHHHHHHHHHHHHHHHHHHHHHHHH     HHH
Human talin1     AVAEQIPLLVQGVRGSQAQPDSPSAQLALIAASQSFLQPGGKMVAAAKASVPTIQDQASA 1017
Mouse talin1     AVAEQIPLLVQGVRGSQAQPDSPSAQLALIAASQSFLQPGGKMVAAAKASVPTIQDQASA 1017
Chicken talin1   VVADQIPMLVQGVRGSQSQPDSPSAQLALIAASQNFLQPGGKMVAAAKATVPTITDQASA 1016
Human talin2     AVADHIPQLVQGVRGSQAQAEDLSAQLALIISSQNFLQPGSKMVSSAKAAVPTVSDQAAA 1019
Mouse talin2     AVADHIPQLVQGVRGSQAQAEDLSAQLALIISSQNFLQPGSKMVSSAKAAVPTVSDQAAA 1019
Chicken talin2   NVADHIPQLVQGVRGSQAQAEDLSAQLALINSSQNFLQPGSKMVASAKAAVPTVTDQAAA 1012
                  **::** *********:*.:. ******* :**.*****.***::***:***: ***:*
                           H17                            H18
                 HHHHHHHHHHHHHHHHHHHHHHHHHH   HHHHHHHHHHHHHHHHHHHHHHHHHHH
Human talin1     MQLSQCAKNLGTALAELRTAAQKAQEACGPLEMDSALSVVQNLEKDLQEVKAAARDGKLK 1077
Mouse talin1     MQLSQCAKNLGTALAELRTAAQKAQEACGPLEMDSALSVVQNLEKDLQEIKAAARDGKLK 1077
Chicken talin1   MQLSQCAKNLAAALAELRTAAQKAQEACGPLEIDSALGLVQSLERDLKEAKAAARDGKLK 1076
Human talin2     MQLSQCAKNLATSLAELRTASQKAHEACGPMEIDSALNTVQTLKNELQDAKMAAVESQLK 1079
Mouse talin2     MQLSQCAKNLATSLAELRTASQKAHEACGPMEIDSALNTVQTLKNELQDAKMAAAESQLK 1079
Chicken talin2   MQLSQCAKNLATSLAELRTASQKAHEACGPMEIDSALNTVQTLKSELQDAKMAAVDGQLK 1072
                 **********.::*******:***:*****:*:****. **.*: :*:: * ** :.:**
                                   H19                             H20
                     HHHHHHHHHHHHHHHHHHHHHHHHHHHHH   HHHHHHHHHHHHHHHHHHHHHHHH
Human talin1     PLPGETMEKCTQDLGNSTKAVSSAIAQLLGEVAQGNENYAGIAARDVAGGLRSLAQAARG 1137
Mouse talin1     PLPGETMEKCTQDLGNSTKAVSSAIAKLLGEIAQGNENYAGIAARDVAGGLRSLAQAARG 1137
Chicken talin1   PLPGETMEKCAQDLGNSTKAVTSAIAHLLGEVAQGNENYTGIAAREVAQALRSLSQAARG 1136
Human talin2     PLPGETLEKCAQDLGSTSKAVGSSMAQLLTCAAQGNEHYTGVAARETAQALKTLAQAARG 1139
Mouse talin2     PLPGETLEKCAQDLGSTSKGVGSSMAQLLTCAAQGNEHYTGVAARETAQALKTLAQAARG 1139
Chicken talin2   PLPGETLEKCAQDLGSTSKAVGSSMAQLLTCAAQGNEHYTGVAARETAQALKTLAQAARG 1132
                 ******:***:****.::*.* *::*:**   *****:*:*:***:.* .*::*:*****
                                     H21                                H22
                 HHH    HHHHHHHHHHHHHHHHHHHHHHHHHHHHH       HHHHHHHHHHHHHHHHH
Human talin1     VAALTSDPAVQAIVLDTASDVLDKASSLIEEAKKAAGHPGDPESQQRLAQVAKAVTQALN 1197
Mouse talin1     VAALTSDPAVQAIVLDTASDVLDKASSLIEEAKKASGHPGDPESQQRLAQVAKAVTQALN 1197
Chicken talin1   VAANSSDPQAQNAMLECASDVMDKANNLIEEARKAVAKPGDPDSQQRLVQVAKAVSQALN 1196
Human talin2     VAASTTDPAAAHAMLDSARDVMEGSAMLIQEAKQALIAPGDAERQQRLAQVAKAVSHSLN 1199
Mouse talin2     VAASTNDPEAAHAMLDSARDVMEGSAMLIQEAKQALIAPGDTESQQRLAQVAKAVSHSLN 1199
Chicken talin2   VAASTTDPVAAHAMLDSARDVMEGSAMLIQEAKQALAAPGDADSQQRLAQVAKAVSHSLN 1192
                 *** :.** .   :*: * **:: :  **:**::*   ***.: ****.******:::**
                                   H23                               H24
                 HHHH       HHHHHHHHHHHHHHHHH         HHHHHHHHHHHHHHHHHHHHHHH
Human talin1     RCVSCLPGQRDVDNALRAVGDASKRLLSDSLPPSTGTFQEAQSRLNEAAAGLNQAATELV 1257
Mouse talin1     RCVSCLPGQRDVDNALRAVGDASKRLLSDLLPPSTGTFQEAQSRLNEAAAGLNQAATELV 1257
Chicken talin1   RCVNCLPGQRDVDAAIRMVGEASKRLLSDSFPPSNKTFQEAQSQLNRAAAGLNQSANELV 1256
Human talin2     NCVNCLPGQKDVDVALKSIGESSKKLLVDSLPPSTKPFQEAQSELNQAAADLNQSAGEVV 1259
Mouse talin2     NCVNCLPGQKDVDVALKSIGEASKKLLVDSLPPSTKPFQEAQSELNQAAADLNQSAGEVV 1259
Chicken talin2   NCVNCLPGQKDVDVALKSIGESSKKLLVDLLPPSSKSFQEAQSELNQAAADLNQSAGEVV 1252
                 .**.*****:*** *:: :*::**:** * :***. .******.**.***.***:* *:*
```

```
                                    H25                             H26
                  HHH   HHHHHHHHHHHHHHHHHHHHHHHHHH   HHHHHHHHHHHHHHHHHHHHHHHHH
Human talin1      QASRGTPQDLARASGRFGQDFSTFLEAGVEMAGQAPSQEDRAQVVSNLKGISMSSSKLLL 1317
Mouse talin1      QASRGTPQDLARASGRFGQDFSTFLEAGVEMAGQAPSQEDRAQVVSNLKGISMSSSKLLL 1317
Chicken talin1    QASRGTPQDLAKSSGKFGQDFNEFLQAGVEMASLSPTKEDQAQVVSNLKSISMSSSKLLL 1316
Human talin2      HATRGQSGELAAASGKFSDDFDEFLDAGIEMAGQAQTKEDQIQVIGNLKNISMASSKLLL 1319
Mouse talin2      HATRGQSGELAAASGKFSDDFDEFLDAGIEMAGQAQTKEDQMQVIGNLKNISMASSKLLL 1319
Chicken talin2    HATRGQSGELAAASGKFSDDFDEFLDAGIEMAGQAQTKEDQIQVIGNLKSISMASSKLLL 1312
                  :*:** . :** :**:*.:**. **:**:***. : ::**: **:.***.***:******
                                          H27                         H28
                  HHHHHH       HHHHHHHHHHHHHHHHHHHHHHH      HHHHHHHHHHHHHHHHHH
Human talin1      AAKALSTDPAAPNLKSQLAAAARAVTDSINQLITMCTQQAPGQKECDNALRELETVRELL 1377
Mouse talin1      AAKALSTDPASPNLKSQLAAAARAVTDSINQLITMCTQQAPGQKECDNALRQLETVRELL 1377
Chicken talin1    AAKALSADPTSPNLKSQLAAAARAVTDSINQLITMCTQQAPGQKECDNALRELETVKELL 1376
Human talin2      AAKSLSVDPGAPNAKNLLAAAARAVTESINQLITLCTQQAPGQKECDNALRELETVKGML 1379
Mouse talin2      AAKSLSVDPGAPNAKNLLAAAARAVTESINQLIMLCTQQAPGQKECDNALRELETVKGML 1379
Chicken talin2    AAKSLSVDPGAPNAKNLLAAAARAVTESINQLITLCTQQAPGQKECDNALRELETVKGML 1372
                  ***:**.** :** *. *********:****** :****************:****: :*
                                          H29                              H30
                  H           HHHHHHHHHHHHHHHHHHHHHHHHH        HHHHHHHHHHHHHHH
Human talin1      ENPVQPINDMSYFGCLDSVMENSKVLGEAMTGISQNAKNGNLPEFGDAISTASKALCGFT 1437
Mouse talin1      ENPVQPINDMSYFGCLDSVMENSKVLGEAMTGISQNAKNGNLPEFGDAIATASKALCGFT 1437
Chicken talin1    ENPTQTVNDMSYFSCLDSVMENSKVLGESMAGISQNAKNSKLPEFGESISAASKALCGLT 1436
Human talin2      DNPNEPVSDLSYFDCIESVMENSKVLGESMAGISQNAKTGDLPAFGECVGIASKALCGLT 1439
Mouse talin2      ENPNEPVSDLSYFDCIESVMENSKVLGESMAGISQNAKTGDLPAFGECVGIASKALCGLT 1439
Chicken talin2    DNPNEPVSDLSYFDCIEGVMENSKALGESMAGISQNAKTGDLLVFGECVGVASKALCGLT 1432
                  :** :.:.*:***.*::.******.***:*:*******...*  **:.:. *******:*
                                                 H31
                  HHHHHHHHHHH       HH   HHHHHHHHHHHHHHHHHHHHH      HHHHHHHHHH
Human talin1      EAAAQAAYLVGVSDPNSQAGQQGLVEPTQFARANQAIQMACQSLGEPGCTQAQVLSAATI 1497
Mouse talin1      EAAAQAAYLVGVSDPNSQAGQQGLVEPTQFARANQAIQMACQSLGEPGCTQAQVLSAATI 1497
Chicken talin1    EAAAQAAYLVGVSDPNSQAGQQGLVDPTQFARANQAIQMACQNLVDPACTQSQVLSAATI 1496
Human talin2      EAAAQAAYLVGISDPNSQAGHQGLVDPIQFARANQAIQMACQNLVDPGSSPSQVLSAATI 1499
Mouse talin2      EAAAQAAYLVGISDPNSQAGHQGLVDPIQFARANQAIQMACQNLVDPGSSPSQVLSAATI 1499
Chicken talin2    EAAAQAAYLVGISDPNSQAGQQGLVDPIQFARANQAIQMACQNLVDPASSPSQVLSAATI 1492
                  ***********:********:****:* **************.* :*..: :********
                  H32                                H33
                  HHHHHHHHHHHHHHHHH    HHHHHHHHHHHHHHHHHHHHHHHHHHHH     HHHHHH
Human talin1      VAKHTSALCNSCRLASARTTNPTAKRQFVQSAKEVANSTANLVKTIKALDGAFTEENRAQ 1557
Mouse talin1      VAKHTSALCNSCRLASARTANPTAKRQFVQSAKEVANSTANLVKTIKALDGDFTEENRAQ 1557
Chicken talin1    VAKHTSALCNTCRLASSRTANPVAKRQFVQPAKEVANSTANLVKTIKALDGAFNEENRER 1556
Human talin2      VAKHTSALCNACRIASSKTANPVAKRHFVQSAKEVANSTANLVKTIKALDGDFSEDNRNK 1559
Mouse talin2      VAKHTSALCNACRIASSKTANPVAKRHFVQSAKEVANSTANLVKTIKALDGDFSEDNRNK 1559
Chicken talin2    VAKHTSALCNACRIASSKTANPVAKRHFVQSAKEVANSTANLVKTIKALDGDFSEENRNK 1552
                  **********:**:**::*:**.***:***.******************** *.*:** :
                       H34                                            H35
                  HHHHHHHHHHHHHHHHHH                 HHHHHHHHHHHHHHHHHHHHHHHHH
Human talin1      CRAATAPLLEAVDNLSAFASNPEFSSIPAQISPEGRAAMEPIVISAKTMLESAGGLIQTA 1617
Mouse talin1      CRAATAPLLEAVDNLSAFASNPEFSSVPAQISPEGRAAMEPIVISAKTMLESAGGLIQTA 1617
Chicken talin1    CRAATAPLIEAVDNLTAFASNPEFATVPAQISPEGRRAMEPIVTSAKTMLESSAGLIQTA 1616
Human talin2      CRIATAPLIEAVENLTAFASNPEFVSIPAQISSEGSQAQEPILVSAKTMLESSSYLIRTA 1619
Mouse talin2      CRIATTPLIEAVENLTAFASNPEFASIPAQISSEGSQAQEPILVSAKTMLESSSYLIRTA 1619
Chicken talin2    CRIATAPLIEAVENLTAFASNPEFVSIPAQISTEGSRAQEPILVSAKTMLESSSLLIKTA 1612
                  ** **:**:***:**:******** ::*****.**  * ***: ********:. **:**
                                       H36                            H37
                  HHHH      HHHHHHHHHHHHHHHHHHHHHHHHH     HHHHHHHHHHHHHHHHHHHH
Human talin1      RALAVNPRDPPSWSVLAGHSRTVSDSIKKLITSMRDKAPGQLECETAIAALNSCLRDLDQ 1677
Mouse talin1      RALAVNPRDPPRWSVLAGHSRTVSDSIKKLITSMRDKAPGQLECETAIAALNSCLRDLDQ 1677
Chicken talin1    RSLAVNPKDPPQWSVLAGHSRTVSDSIKKLITNMRDKAPGQRECDEAIDVLNRCMREVDQ 1676
Human talin2      RSLAINPKDPPTWSVLAGHSHTVSDSIKSLITSIRDKAPGQRECDYSIDGINRCIRDIEQ 1679
Mouse talin2      RSLAINPKDPPTWSVLAGHSHTVSDSIKSLITSIRDKAPGQRECDYSIDGINRCIRDIEQ 1679
Chicken talin2    RSLAINPKDPPTWSVLAGHSHTVSDSIKSLITSIRDKAPGQRECDYSIDGINRCIRDIEQ 1672
                  *:**:**:*** ********:*******.***.:******* **: :*  :* *:*:::*
```

```
                                             H38
                 HHHHHHHH       HHHHHHHHHHHHHHHHHHHHHHHHHH     HHHHHHHHHHHHHH
Human talin1     ASLAAVSQQLAPREGISQEALHTQMLTAVQEISHLIEPLANAARAEASQLGHKVSQMAQY 1737
Mouse talin1     ASLAAVSQQLAPREGISQEALHTQMLTAVQEISHLIEPLASAARAEASQLGHKVSQMAQY 1737
Chicken talin1   ASLAAISQQLAPREGISQEALHNQMITAVQEINNLIEPVASAARAEASQLGHKVSQMAQY 1736
Human talin2     ASLAAVSQSLATRDDISVEALQEQLTSVVQEIGHLIDPIATAARGEAAQLGHKVTQLASY 1739
Mouse talin2     ASLAAVSQSLATRDDISVEALQEQLTSVVQEIGHLIDPIATAARGEAAQLGHKVTQLASY 1739
Chicken talin2   ASLAAVSQSLATRDDISVEALQEQLTSVVQEIGHLIDPIATAARGEAAQLGHKVTQLASY 1732
                 *****:**.**.*:.** ***: *: :.****.:**:*:*.***.**:******:*:*.*
                     H39                        H40
                 HHHHHHHHHHHHHHH   HHHHHHHHHHHHHHHHHHHHHHHHHH     HHHHHHHHHHH
Human talin1     FEPLTLAAVGAASKTLSHPQQMALLDQTKTLAESALQLLYTAKEAGGNPKQAAHTQEALE 1797
Mouse talin1     FEPLTLAAVGAASKTLSHPQQMALLDQTKTLAESALQLLYTAKEAGGNPKQAAHTQEALE 1797
Chicken talin1   FEPLILAAIGAASKTPNHQQQMNLLDQTKTLAESALQMLYTAKEAGGNPKQAAHTQEALE 1796
Human talin2     FEPLILAAVGVASKILDHQQQMTVLDQTKTLAESALQMLYAAKEGGGNPK-AQHTHDAIT 1798
Mouse talin2     FEPLILAAVGVASKMLDHQQQMTVLDQTKTLAESALQMLYAAKEGGGNPKQAQHTHDAIT 1799
Chicken talin2   FEPLVLAAVGVASKTLDHQQQMTVLDQSKTLAESALQMLYAAKEGGGNPKLDESTPADPK 1792
                 **** ***:*.***  .* *** :***:*********:**:***.*****    *
                         H41                    H42
                 HHHHHHHHHHHHHHHHHHHHHH   HHHHHHHHHHHHHHH           HHHHHHHHH
Human talin1     EAVQMMTEAVEDLTTTLNEAASAAGVVGGMVDSITQAINQLDEGPMGEPEGSFVDYQTTM 1857
Mouse talin1     EAVQMMTEAVEDLTTTLNEAASAAGVVGGMVDSITQAINQLDEGPMGDPEGSFVDYQTTM 1857
Chicken talin1   EAVQMMKEAVEDLTTTLNEAASAAGVVGGMVDSITQAINQLDEGPMGEPEGTFVDYQTTM 1856
Human talin2     EAAQLMKEAVDDIMVTLNEAASEVGLVGGMVDAIAEAMSKLDEGTPPEPKGTFVDYQTTV 1858
Mouse talin2     EAAQLMKEAVDDIMVTLNEAASEVGLVGGMVDAIAEAMSKLDEGTPPEPKGTFVDYQTTV 1859
Chicken talin2   GTFVDYQTTVVKYSKAIAITAQEMMFCECLIIMTLIKHACLSFLTEFLLENNSLTRQAFF 1852
                  :      :* .   ::  :*.   .   ::         *.  .    :.. :  *: .
                     H43                           H44
                 HHHHHHHHHHHHHHHH      HHHHHHHHHHHHHHHHHHHHHHHHHHH   HHHHHHHH
Human talin1     VRTAKAIAVTVQEMVTKSNTSPEELGPLANQLTSDYGRLASEAKPAAVAAENEEIGSHIK 1917
Mouse talin1     VRTAKAIAVTVQEMVTKSNTSPEELGPLANQLTSDYGRLASQAKPAAVAAENEEIGAHIK 1917
Chicken talin1   VKTAKAIAVTVQEMVTKSTTNPDELGILANQLTNDYGQLAQQAKPAALTAENEEIGSHIK 1916
Human talin2     VKYSKAIAVTAQEMMTKSVTNPEELGGLASQMTSDYGHLAFQGQMAAATAEPEEIGFQIR 1918
Mouse talin2     VKYSKAIAVTAQEMMTKSVTNPEELGGLASQMTTDYGHLALQGQMAAATAEPEEIGFQIR 1919
Chicken talin2   LTSS----------MTKSVTNPEELGGLASQMTNDYGHLALQGRMAAATAEPEEIGFQIK 1902
                 :  :          :*** *.*:*** **.*:*.***:** :.: ** :** **** :*:
                    H45                                 H46
                 HHHHHHHHHHHHHHHHHHHHHH    HHHHHHHHHHHHHHHHHHHHHHHHHHH     HH
Human talin1     HRVQELGHGCAALVTKAGALQCSPSDAYTKKELIECARRVSEKVSHVLAALQAGNRGTQA 1977
Mouse talin1     HRVQELGHGCSALVTKAGALQCSPSDVYTKKELIECARRVSEKVSHVLAALQAGNRGTQA 1977
Chicken talin1   RRVQELGHGCAALVTKAGALQCSPSDAYTKKELIESARKVSEKVSHVLAALQAGNRGTQA 1976
Human talin2     TRVQDLGHGCIFLVQKAGALQVCPTDSYTKRELIECARAVTEKVSLVLSALQAGNKGTQA 1978
Mouse talin2     TRVQDLGHGCIFLVQKAGALQVCPTDSYTKRELIECARSVTEKVSLVLSALQAGNKGTQA 1979
Chicken talin2   TRVQELGHGCIFLVQKAGALQICPTDSYTKRELIECARAVTEKVSLVLSALQAGNKGTQA 1962
                  ***:*****  ** ****** .*:* ***:****.** *:**** **:******:****
                        H47                                  H48
                 HHHHHHHHHHHHHHHHHHHHHHH           HHHHHHHHHHHHHHHHHHHHHHH
Human talin1     CITAASAVSGIIADLDTTIMFATAGTLNREGTETFADHREGILKTAKVLVEDTKVLVQNA 2037
Mouse talin1     CITAASAVSGIIADLDTTIMFATAGTLNREGAETFADHREGILKTAKVLVEDTKVLVQNA 2037
Chicken talin1   CITAASAVSGIIADLDTTIMFATAGTLNRENSETFADHREGILKTAKALVEDTKVLVQNA 2036
Human talin2     CITAATAVSGIIADLDTTIMFATAGTLNAENSETFADHRENILKTAKALVEDTKLLVSGA 2038
Mouse talin2     CITAATAVSGIIADLDTTIMFATAGTLNAENGETFADHRENILKTAKALVEDTKLLVSGA 2039
Chicken talin2   CITAASAVSGIIADLDTTIMFATAGTLNAENNESFADHRENILKTAKALVEDTKLLVSGA 2022
                 *****:********************** *. *:******.******.******:**..*
                                 H49                                 H50
                    HHHHHHHHHHHHHHHHHHHHHHHHHHHH       HHHHHHHHHHHHHHHHHHHHHH
Human talin1     AGSQEKLAQAAQSSVATITRLADVVKLGAASLGAEDPETQVVLINAVKDVAKALGDLISA 2097
Mouse talin1     AGSQEKLAQAAQSSVATITRLADVVKLGAASLGAEDPETQVVLINAVKDVAKALGDLISA 2097
Chicken talin1   TASQEKLAQAAQSSVSTITRLAEVVKLGAASLGSEDPETQVVLINAVKDVAKALGDLIGA 2096
Human talin2     ASTPDKLAQAAQSSAATITQLAEVVKLGAASLGSDDPETQVVLINAIKDVAKALSDLISA 2098
Mouse talin2     ASTPDKLAQAAQSSAATITQLAEVVKLGAASLGSNDPETQVVLINAIKDVAKALSDLIGA 2099
Chicken talin2   ASSQDKLAQAAQSSANTITQLAEVVKLGAASLGSDDPETQVVLINAIKDVAKALSDLIGA 2082
                 :.: :*********. ***:**:**********::***********:*******.***.*
```

```
                                       H51                           H52
                HHHHHH      HHHHHHHHHHHHHHHHHHHHHHH    HHHHHHHHHHHHHHHHHHHHH
Human talin1    TKAAAGKVGDDPAVWQLKNSAKVMVTNVTSLLKTVKAVEDEATKGTRALEATTEHIRQEL 2157
Mouse talin1    TKAAAGKVGDDPAVWQLKNSAKVMVTNVTSLLKTVKAVEDEATKGTRALEATTEHIRQEL 2157
Chicken talin1  TKAAAGKAGDDPAVYQLKNSAKVMVTNVTSLLKTVKAVEDEATKGTRALEATIEHIRQEL 2156
Human talin2    TKGAASKPVDDPSMYQLKGAAKVMVTNVTSLLKTVKAVEDEATRGTRALEATIECIKQEL 2158
Mouse talin2    TKGAASKPADDPSMYQLKGAAKVMVTNVTSLLKTVKAVEDEATRGTRALEATIEYIKQEL 2159
Chicken talin2  TKGAASKPADDPSMYQLKGAAKVMVTNVTSLLKTVKAVEDEATRGTRALEATIEYIKQEL 2142
                **.**.*  ***:::***.:***********************:******** * *:***
                                        H53                          H54
                HHH           HHHHHHHHHHHHHHHHHHHHH     HHHHHHHHHHHHHHHHHHHH
Human talin1    AVFCSPEPPAKTSTPEDFIRMTKGITMATAKAVAAGNSCRQEDVIATANLSRRAIADMLR 2217
Mouse talin1    AVFCSPEPPAKTSTPEDFIRMTKGITMATAKAVAAGNSCRQEDVIATANLSRRAIADMLR 2217
Chicken talin1  AVFSSPVPPAQVSTPEDFIRMTKGITMATAKAVAAGNSCRQEDVIATANLSRRAIADMLR 2216
Human talin2    TVFQSKDVPEKTSSPEESIRMTKGITMATAKAVAAGNSCRQEDVIATANLSRKAVSDMLT 2218
Mouse talin2    TVFQSKDIPEKTSSPEESIRMTKGITMATAKAVAAGNSCRQEDVIATANLSRKAVSDMLI 2219
Chicken talin2  TVFQSNEVPEKTSSPEESIRMTKGITMATAKAVAAGNSCRQEDVIATANLSRKAVADMLT 2202
                :** *   * :.*:**: **********************************:*::***
                                         H55                             H56
                HHHHH        HHHHHHHHHHHHHHHHHHHHHHHHHHH       HHHHHHHHHHHHH
Human talin1    ACKEAAYHPEVAPDVRLRALHYGRECANGYLELLDHVLLTLQKPSPELKQQLTGHSKRVA 2277
Mouse talin1    ACKEAAFHPEVAPDVRLRALHYGRECANGYLELLDHVLLTLQKPNPDLKQQLTGHSKRVA 2277
Chicken talin1  ACKEAAYHPEVSADVRQRALRFGKECADGYLELLEHVLVILQKPTHELKQQLAGYSKRVA 2276
Human talin2    ACKQASFHPDVSDEVRTRALRFGTECTLGYLDLLEHVLVILQKPTPEFKQQLAAFSKRVA 2278
Mouse talin2    ACKQASFYPDVSEEVRTRALRYGTECTLGYLDLLEHVLVILQKPTPELKHQLAAFSKRVA 2279
Chicken talin2  ACKQASYHQEVSEEVRERALRFGTECTLGYLELLEHVLLILQKPTPELKHQLAAFSKRVA 2262
                ***:*::: :*: :** ***::* **: ***:**:***: ****. ::*:**:..*****
                                                     H57
                HHHHHHHHHHHH           HHHHHHHHHHHHHHHHHHHHHHHHHHHHH
Human talin1    GSVTELIQAAEAMKGTEWVDPEDPTVIAENELLGAAAAIEAAAKKLEQLKPRAKPKEADE 2337
Mouse talin1    GSVTELIQAAEAMKGTEWVDPEDPTVIAENELLGAAAAIEAAAKKLEQLKPRAKPKEADE 2337
Chicken talin1  SSVTELIQAAEAMKGTEWVDPEDPTVIAENELLGAAAAIEAAAKKLEQLKPRAKPKQADE 2336
Human talin2    GAVTELIQAAEAMKGTEWVDPEDPTVIAETELLGAAASIEAAAKKLEQLKPRAKPKQADE 2338
Mouse talin2    GAVTELIQAAEAMKGTEWVDPEDPTVIAETELLGAAASIEAAAKKLEQLKPRAKPKQADE 2339
Chicken talin2  SAVTELIQSAEAMKGTEWVDPEDPTVIAETELLGAAASIEAAAKKLEQLKPRAKPKQADE 2322
                .:******:********************.*******:******************:***
                                   H58
                   HHHHHHHHHHHHHHHHHHHHHHHHHHHHHHHHH       HHHHHHHHHHHHHHHHH
Human talin1    SLNFEEQILEAAKSIAAATSALVKAASAAQRELVAQGKVGAIPANALDDGQWSQGLISAA 2397
Mouse talin1    SLNFEEQILEAAKSIAAATSALVKAASAAQRELVAQGKVGAIPANALDDGQWSQGLISAA 2397
Chicken talin1  SLDFEEQILEAAKSIAAATSALVKAASAAQRELVAQGKVGVIPANAVDDGQWSQGLISAA 2396
Human talin2    TLDFEEQILEAAKSIAAATSALVKSASAAQRELVAQGKVGSIPANAADDGQWSQGLISAA 2398
Mouse talin2    TLDFEEQILEAAKSIAAATSALVKSASAAQRELVAQGKVGSIPANAADDGQWSQGLISAA 2399
Chicken talin2  TLDFEEQILEAAKSIAAATSALVKSASAAQRELVAQGKVGAIPANAADDGQWSQGLISAA 2382
                :*:*********************:*************** ***** *************
                 H59                               H60
                HHHHHHHHHHHHHHHHHH     HHHHHHHHHHHHHHHHHHHHHHHHH     HHHHHHH
Human talin1    RMVAAATNNLCEAANAAVQGHASQEKLISSAKQVAASTAQLLVACKVKADQDSEAMKRLQ 2457
Mouse talin1    RMVAAATNNLCEAANAAVQGHASQEKLISSAKQVAASTAQLLVACKVKADQDSEAMKRLQ 2457
Chicken talin1  RMVAAATNNLCEAANAAVQGHASEEKLISSAKQVAASTAQLLVACKVKADHDSEAMKRLQ 2456
Human talin2    RMVAAATSSLCEAANASVQGHASEEKLISSAKQVAASTAQLLVACKVKADQDSEAMRRLQ 2458
Mouse talin2    RMVAAATSSLCEAANASVQGHASEEKLISSAKQVAASTAQLLVACKVKADQDSEAMKRLQ 2459
Chicken talin2  RMVAAATSNLCEAANASVQGHASEEKLISSAKQVAASTAQLLVACKVKADHDSEAMRRLQ 2442
                *******..*******:******:**************************:*****:***
                      H61                                            H62
                HHHHHHHHHHHHHHHHHHHHHHHHH           HHHHHHHHHHHHHHHHHHHHHHHH
Human talin1    AAGNAVKRASDNLVKAAQKAAAFEEQENETVVVKEKMVGGIAQIIAAQEEMLRKERELEE 2517
Mouse talin1    AAGNAVKRASDNLVKAAQKAAAFEDQENETVVVKEKMVGGIAQIIAAQEEMLRKERELEE 2517
Chicken talin1  AAGNAVKRASDNLVKAAQKAAAFQDHD-ETVVVKEKMVGGIAQIIAAQEEMLRKERELEE 2515
Human talin2    AAGNAVKRASDNLVRAAQK-AAFGKADDDDVVVKTKFVGGIAQIIAAQEEMLKKERELEE 2517
Mouse talin2    AAGNAVKRASDNLVRAAQK-AAFGKADDDDVVVKTKFVGGIAQIIAAQEEMLKKERELEE 2518
Chicken talin2  AAGNAVKRASDNLVRAAQK-AAFGKAEDDDVVVKTKFVGGIAQIIAAQEEMLKKERELEE 2501
                **************:**** *** . : : **** *:***************:*******
```

```
                 HHHHHHH  HH      HHH
Human talin1     ARKKLAQIRQQQYKFLPSELRDEH-- 2541
Mouse talin1     ARKKLAQIRQQQYKFLPSELRDEH-- 2541
Chicken talin1   ARKKLAMIRQQQYKFLPTELRDEEQN 2541
Human talin2     ARKKLAQIRQQQYKFLPTELREDEG- 2542
Mouse talin2     ARKKLAQIRQQQYKFLPTELREDEG- 2543
Chicken talin2   ARKKLAQIRQQQYKFLPTELREDEG- 2526
                 ****** **********:***::.
```
