# Supplementary material for: How Force Might Activate Talin's Vinculin Binding Sites: SMD Reveals a Structural Mechanism
Source: PLoS Comput Biol. 2008 Feb 15;4(2):e24. doi: 10.1371/journal.pcbi.0040024 (PMC2242828; doi:10.1371/journal.pcbi.0040024)
Supplement: Supplementary file 2 [file pcbi.0040024.st001.doc]

# Supporting Information

# Supporting Table

### Table S1 - Protein fragments used in this study

| **Protein**  **(residues)** | **Total number of atoms**  **(protein atoms)** | **Added ions** | **Water box dimensions**  **(x, y, z) (Å)** | **PDB ID** |
| --- | --- | --- | --- | --- |
| H1-H12  (486–889) | 94495  (5795) | 17 Na^+a^ | 170, 83, 71 | 1XWX^b^ |
| H1-H9  (486-782) | 89530  (4243) | 12 Na^+^ | 111, 115, 74 | 1SJ8 |
| H2-H8  (523–757) | 115790  (3358) | 7 Na^+^ | 123, 136, 77 | 1XWX |
| H9-H12  (755–889) | 82221  (1830) | 6 Na^+^ | 92, 91, 103 | 1XWX |
| H1-H12  (talin2) | 111073  (5918) | 20 Na^+^ | 164, 88, 74 | _^c^ |

^a^18 Na^+^-ions in the case of forms containing mutation A744D

^b^PDB theoretical models section

^c^Homology model based on [PDB: 1XWX]
